# The Determinants of Long‐Term Sustainment of Healthy Food Retail Environment Changes: A Systematic Review

**DOI:** 10.1111/obr.70143

**Published:** 2026-04-28

**Authors:** Bettina Backman, Cédric N. H. Middel, Serene Yoong, Adyya Gupta, Noor Imad, Vicki Brown, Miranda R. Blake

**Affiliations:** ^1^ Global Centre for Preventive Health and Nutrition, Institute for Health Transformation, School of Health and Social Development, Faculty of Health Deakin University Geelong Australia; ^2^ Department of Epidemiology & Data Science Amsterdam UMC Amsterdam The Netherlands; ^3^ Amsterdam Public Health Research Institute Amsterdam The Netherlands; ^4^ Upstream Team Amsterdam The Netherlands; ^5^ College of Health, Medicine and Wellbeing University of Newcastle Callaghan NSW Australia; ^6^ Department of Nursing and Allied Health, School of Health Sciences Swinburne University Melbourne Australia; ^7^ Deakin Health Economics (DHE), Institute for Health Transformation Deakin University Geelong Australia

**Keywords:** consumer behavior, food services, food supply, health promotion, implementation science, public health

## Abstract

Healthy food retail (HFR) interventions utilize marketing strategies (e.g., product availability, placement, pricing, and promotion) to create food outlet (e.g., grocery store, food service) environments that support consumers in making healthier food purchases. To ensure ongoing population health benefits, these strategies must be sustained over time. This systematic review aimed to explore the determinants of sustained HFR environment changes (e.g., sociopolitical context, retailer‐related factors). Six databases were searched up to September 12, 2024. Eligible articles followed up on HFR environment changes for at least 2 years and identified factors influencing sustained HFR changes. Articles were thematically synthesized using the Integrated Sustainability Framework, and the findings for each construct were presented as frequency counts. Risk of bias was assessed using the Mixed Methods Appraisal Tool. Fifteen articles met the inclusion criteria, with studies undertaken in grocery stores or small stores (*n* = 8), school canteens (*n* = 3), healthcare settings (*n* = 2), and other settings (*n* = 2). The most common domains identified were “processes” (e.g., “fidelity, monitoring, and enforcement”) and “interorganizational factors” (e.g., “external partners, support, and resources”). Sustainment‐related findings were often reported as secondary outcomes in study results, limiting the comprehensiveness of the review and highlighting the need for more long‐term evaluation of HFR interventions.

**Abbreviations**
ERIC: Expert Recommendations for Implementing Change
HFR: healthy food retail
ISF: Integrated Sustainability Framework
MMAT: Mixed Methods Appraisal Tool
PRISMA: Preferred Reporting Items for Systematic Reviews and Meta‐analyses
START: Systems Thinking Approach for Retail Transformation
US: the United States

## 1 Background

In‐store food environments where consumers purchase food and drinks play an essential role in shaping dietary behaviors and population health outcomes [1]. Strong evidence demonstrates that in‐store product availability, placement, pricing strategies, and promotional activities (the “4Ps of marketing”) can impact the selection of food and drinks purchased by consumers [1]. Current in‐store food environments across the globe predominantly promote unhealthy food choices, such as foods high in energy, saturated fat, sugar, and salt and low in dietary fiber [2]. Unhealthy food environments, characterized by in‐store marketing favoring unhealthy foods, have been associated with an increased risk of obesity, diabetes, cardiovascular diseases, and other chronic health conditions [2, 3]. Conversely, in‐store food environments that favor healthier food options, such as fruit and vegetables, can contribute to improved dietary behaviors and overall population health [1, 4, 5].

Previous systematic reviews suggest that utilizing the “4Ps of marketing” strategies has the potential to improve the healthiness of consumers' food purchases [1, 4, 5]. Healthy food retail (HFR) interventions often aim to change the in‐store food environment by implementing strategies that promote healthier food products utilizing one or more of the “4Ps of marketing” strategies [4]. Multiple factors can affect the successful implementation of these strategies (e.g., retailer motivation and skills, customer purchasing behavior) across different food retail settings [6, 7]. For example, the Systems Thinking Approach for Retail Transformation (START) map identifies key factors influencing the implementation of HFR interventions in public food outlets, including factors related to retailers, customers (consumers who purchase items from a specific outlet), governing organizations, the in‐store food environment, and the broader environment [6]. These factors largely overlap with those identified in systematic reviews of private food retail settings [7, 8]. Implementation strategies addressing these factors can support retailers to improve the healthiness of their in‐store food environment [9]. For example, evidence shows that implementation strategies (e.g., training) targeting determinants of implementation (e.g., retailer skills) are effective in driving healthy changes in in‐store food environments [10, 11]. Continuous maintenance or “sustainment” of healthy changes in food outlets is required to support long‐term changes in consumer purchasing behavior. “Sustainment” includes food outlets' capacity to maintain HFR strategies that continuously support customers in choosing healthier food options [9, 12, 13].

While factors influencing sustainment of HFR interventions are likely to overlap with factors affecting implementation of HFR interventions [14], there is a lack of evidence regarding *whether* and *how* in‐store food environment changes are sustained in the long term (at least 2 years post initial‐implementation) [4, 13]. A previous systematic review of supermarket‐based interventions found that HFR interventions are rarely followed‐up after implementation support and funding has ended [4]. While there is a growing body of evidence on factors associated with effective *implementation* of HFR interventions [7, 13, 15] and sustainment of health promotion interventions more broadly [16, 17, 18, 19], there appears to be limited evidence on the determinants of *sustained* HFR interventions specifically. A systematic review by Houghtaling et al. [8] identified that individual‐level factors (e.g., retailer skills and knowledge) are likely to influence retailer willingness and ability to implement and sustain HFR interventions in food retail outlets. However, this review focused specifically on store owner perspectives in the United States (US) and looked at the determinants of implementation and sustainment simultaneously. An umbrella review by Gupta et al. (2022) [13] described factors influencing implementation, sustainment, and scalability of HFR interventions using a socio‐ecological perspective. The review found very limited evidence on factors specifically influencing sustainment and did not explore how these factors align with sustainment‐specific theories and frameworks (e.g., Integrated Sustainability Framework (ISF) [20]). The ISF [20] provides a comprehensive, evidence‐based approach by integrating factors considered influential in sustaining health interventions across various contexts [20]. Using an empirical, sustainment‐specific framework offers an evidence‐based and structured approach to identifying the factors that influence long‐term intervention success, while also helping to understand how the sustainment of HFR interventions may differ from other health interventions [9, 20].

To our knowledge, there are no reviews that have explicitly synthesized the factors associated with sustainment of HFR interventions across different food retail contexts. Determining factors associated with sustained HFR environment changes would allow for the identification and development of strategies that can help food retailers to improve the healthiness of food offered and purchased in their outlets in the long term. This, in turn, could help sustain improvements in population health. Our systematic review aimed to comprehensively identify and synthesize factors that influence (facilitate or hinder) the sustainment of HFR environment changes in settings where consumers commonly make food purchases. We addressed the following research questions: (1) What *determinants* are associated with the long‐term sustainment (at least 2 years post initial implementation) of food retail environment changes aimed at improving the healthiness of food and drinks purchased by consumers? (2) Do the *determinants* associated with sustainment vary by retail setting type (e.g., independent grocery stores, for‐profit school canteens)? (3) Do the implementation and sustainment *strategies* used in the reported interventions address the identified determinants of sustainment?

## 2 Methods

This review was conducted in accordance with the Preferred Reporting Items for Systematic Reviews and Meta‐analyses (PRISMA) guidelines [21] (see Additional file I) and JBI Manual for Evidence Synthesis [22] and was prospectively registered in PROSPERO (CRD42023451390) [23].

### 2.1 Eligibility Criteria

Articles were eligible for inclusion if they reported on studies examining the sustainment of HFR changes (e.g., changes in product availability, promotional materials) in consumer‐facing food environments at least 2 years post initial implementation and identified any determinants (barriers and facilitators) of sustained changes. The article eligibility and selection criteria are summarized in the Table 1. HFR changes were defined as changes aimed at improving the healthiness of food and/or drinks purchased by food outlet customers (any population).

**TABLE 1:** PICOS criteria guiding the search and screening for a systematic review on determinants of sustained healthy food retail changes.

PICOS	Inclusion	Exclusion
Population and context	Population context: food outlets Consumer‐facing *food retail*, including both *grocery* and *food service outlets* (e.g., supermarkets, cafés, school/workplace canteens, restaurants) where there is a direct exchange of money for food Private (e.g., supermarket) or public settings (e.g., hospital cafés) Physical or online ‘in‐store’ food environment Population context: customers Customers of the food outlets can represent any population group Study participants Any key informants relevant to food retail or food service outlets	Food services embedded within other services (e.g., catering, in‐patient hospital food service, school lunch, or breakfast programs) Charitable food organizations (e.g., food banks) External food environment (e.g., density of fast‐food outlets versus healthy food outlets)
Intervention(s), exposure(s)	*Healthy food retail interventions* that aim to make changes in the in‐store (or online) food environment (e.g., product availability, placement, pricing, or promotion) to improve the healthiness of consumer purchases Food environment changes can result from various intervention types, such as policy interventions, interventionist‐led programs, or multicomponent interventions	Interventions that do not aim to make changes in the in‐store (or online) food environment to improve the healthiness of consumer purchases
Comparator(s)/control	No restrictions.	
Primary outcome(s)	Determinants of sustained changes Factors (barriers or facilitators) associated with the *sustained* healthy in‐store food environment changes (product availability, placement, price, promotion). These can be related (but not limited) to the domains included in the Integrated Sustainability Framework [20] and Systems Thinking Approach for Retail Transformation map [6]: &cir Outer context (e.g., socio‐political environment) &cir Inner context (e.g., capacity, resources) &cir Retailers (e.g., motivation and skills) &cir Customers (e.g., purchasing behavior and needs) &cir Intervention (e.g., cost‐benefits) &cir Processes (e.g., training, monitoring)	Studies not reporting on any factors associated with *sustained* healthy in‐store food environment changes
Types of study	Studies examining the *sustainment (*i.e.*, continuation) and/or determinants of sustainment* of healthy food retail strategies (healthy changes in product availability, placement, pricing, or promotion) implemented in the in‐store (or online) food environment Evidence of sustainment was defined as qualitative or quantitative reporting of continued (or discontinued) implementation of healthy food retail strategies, assessed at least 2 years after the initial implementation period (as reported in the included studies) Original research of any design (quantitative or qualitative), including cross sectional and longitudinal studies	Study protocols Editorials Systematic reviews or systematic review‐like study designs

### 2.2 Search Strategy and Information Sources

An initial search of MEDLINE was conducted to identify relevant keywords in titles, abstracts, and subject descriptors. Identified keywords and synonyms were then used to develop a search strategy with support from a Deakin University health librarian, focusing on four key hedge terms: (1) “food retail outlets,” (2) “food products,” (3) “healthy food retail intervention,” and (4) “determinants of sustainment.” These search terms were based on previous reviews and studies focusing on HFR interventions [7, 13, 15] and reviews focusing on the sustainment of health promotion interventions [18]. A comprehensive literature search was conducted by the lead author across six databases (MEDLINE, EMBASE, CENTRAL, CINAHL, Global Health, and Scopus) in August 2023 and then rerun on September 12, 2024, to capture the most recent evidence. Articles published in English were searched with no date limits. Google Scholar was used to identify additional publications, with the first 100 results included in the article screening. Finally, reference lists of the articles and publications citing the included articles were manually searched. The full search strategy is available in Additional file II.

### 2.3 Article Selection Process

The screening process was facilitated using the Covidence systematic review software [24]. Double independent screening of all titles and abstracts was performed by pairs of review authors (BB, NI, CNHM). Full‐text screening of manuscripts was also conducted in duplication (BB, NI, CNHM, VB, SY). A screening checklist was developed to guide the screening process (Additional file III). Any discrepancies between reviewers regarding article eligibility were resolved through author consensus or by consulting a third reviewer (SY, MB) where required.

### 2.4 Quality Assessment

Included articles were assessed by pairs of independent authors (BB, CNHM, and AG) using the Mixed‐Methods Appraisal Tool (MMAT) [25]. This tool was selected as it is designed to critically appraise different study designs using a single tool with different sets of questions for each study type [25]. The tool consists of two screening questions and five specific questions per study design, with response options of “yes,” “no,” and “cannot tell.” For this review, questions related to qualitative, quantitative descriptive, and mixed‐method studies were included (Additional file IV). Any discrepancies between reviewers were resolved by consensus.

### 2.5 Data Extraction

Double data extraction from the included articles was independently conducted in Covidence by authors BB, CNHM, and AG using the data extraction template prepared for this review (Additional file V). The lead author reviewed the extracted data to ensure its accuracy and completeness. The extracted information included publication and intervention details, study design, type of setting, participants, sample size, in‐store food environment changes, implementation framework used (if applicable), initial and ongoing support provided, intervention and follow‐up durations, and data collection methods relevant to determinants of sustainment (e.g., interviews, surveys). Outcomes of the interventions reported in the included articles were not extracted, as the aim of this review was to identify determinants of sustainment rather than evaluate intervention effectiveness or outcomes. Illustrative quotes representing identified factors associated with sustained HFR changes were extracted from the included papers.

### 2.6 Data Analysis and Synthesis

A thematic approach was used for data synthesis to allow for the convergent integration of qualitative and quantitative findings related to the determinants of sustained HFR changes (“convergent integrated” approach) [22]. All studies were included in the data synthesis, and the critical appraisal results were used to assess the risk of bias in the included studies.

#### 2.6.1 Determinants of Sustainment

Illustrative quotes extracted from the results section of included articles were thematically analyzed to identify determinants of sustained HFR environment changes. Using quotes allowed for transforming quantitative data into a qualitative format [22]. The coding of the quotes was completed by pairs of reviewers BB, CNHM, and AG, with any discrepancies resolved by consensus. The number of articles reporting each factor associated with sustained HFR changes was reported as a frequency count. The strength of each factor was based on the number of studies reporting each factor [26].

The coding of illustrative quotes used a modified version of the ISF [20], with adaptations for food retail context using the START map (Table 3) [6]. The ISF provides a comprehensive, evidence‐based approach by integrating factors considered influential in sustaining health interventions across various contexts [20]. For example, this framework has been recently used in a systematic review by Shoesmith et al. [18] exploring determinants of sustained health interventions in education and childcare settings. The START map focuses on factors influencing the *implementation* of healthy changes in food outlets [6]; thus, it was primarily used to offer a food retail perspective on the determinants likely to influence sustained HFR changes. Six main domains were derived by combining the ISF and START map: (i) outer and (ii) inner contextual factors, (iii) processes and (iv) characteristics of the interventionist (retailer), (v) population (customers), and (vi) intervention [6, 20]. Any factors that could not be placed in any of the domains or categories within these domains were placed separately in the “other” category and were inductively coded reflecting on the review questions and background literature. The framework was updated after the first round of coding to include any new domains or factors identified.

#### 2.6.2 Implementation and Sustainment Support

Any initial and ongoing support described in the included articles was coded by pairs of reviewers (BB, CNHM, and AG) using the sustainment‐explicit Expert Recommendations for Implementing Change (ERIC) glossary. The ERIC glossary was previously developed to help researchers and practitioners identify and select evidence‐based strategies to support adoption, implementation, and maintenance of health interventions [14]. The identified strategies were mapped to the determinants of sustained changes by the lead author and reviewed by co‐authors during manuscript preparation.

## 3 Results

### 3.1 Article Selection

The article selection process is outlined in Figure 1. A total of 7922 unique titles and abstracts were screened for eligibility, of which 515 articles were included in the full‐text screening. Fifteen studies were identified for inclusion [27, 28, 29, 30, 31, 32, 33, 34, 35, 36, 37, 38, 39, 40, 41].

**FIGURE 1:**
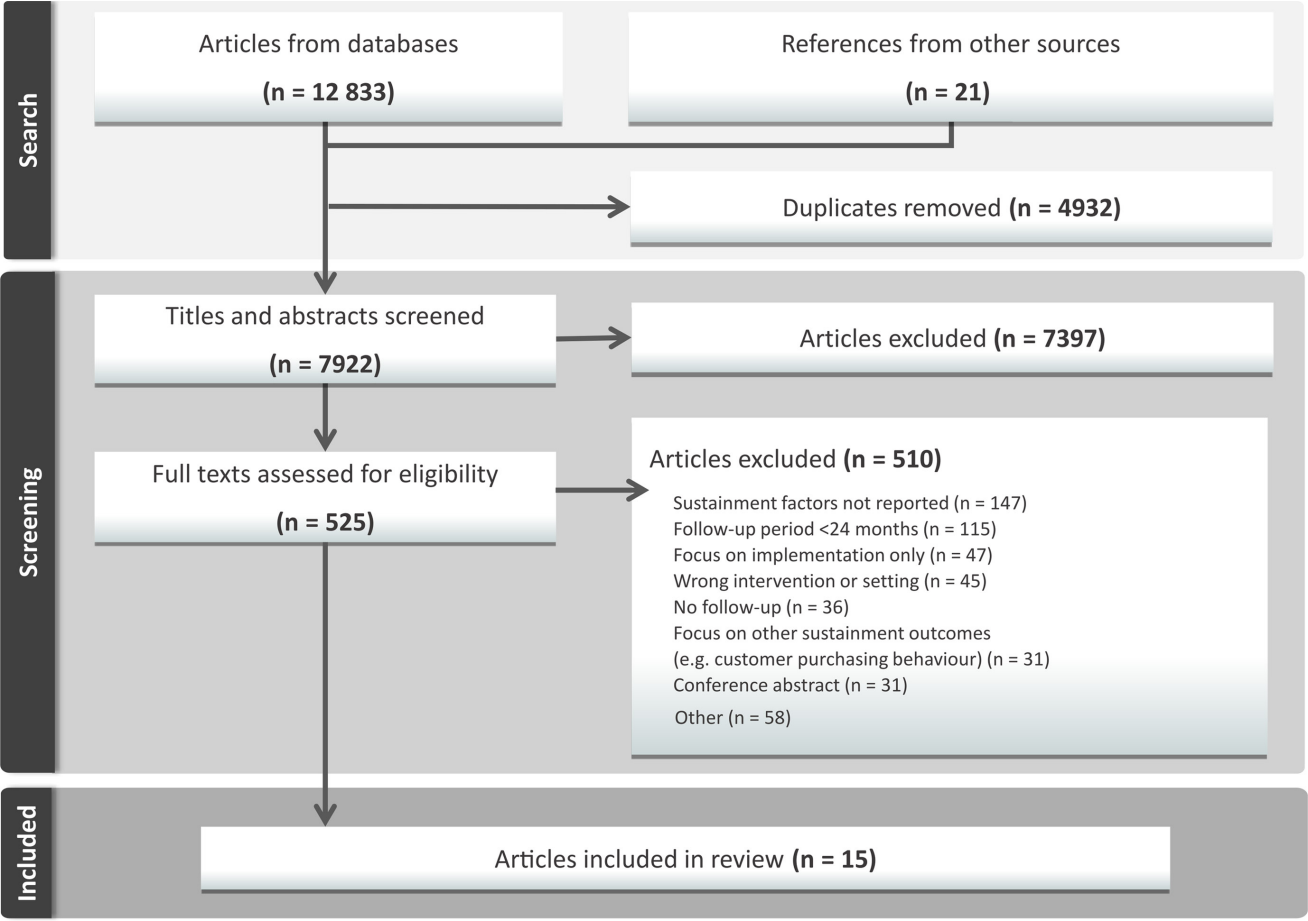
Preferred Reporting Items for Systematic Reviews and Meta‐analyses (PRISMA) flow diagram representing article selection and inclusion process.

### 3.2 Article characteristics

The characteristics of the included articles are summarized in Table 2. Articles were published between 2012 and 2024. Studies were conducted in five high‐income countries: the United States (*n* = 6) [30, 31, 33, 34, 38, 39], Australia (*n* = 3) [27, 29, 40], the United Kingdom (UK) (*n* = 2) [35, 37], Canada (*n* = 3) [28, 32, 36], and New Zealand [41]. Most articles reported on studies that employed a qualitative design (*n* = 9) [27, 28, 31, 32, 34, 35, 36, 40, 41], followed by mixed‐method design (*n* = 5) [30, 33, 37, 38, 39] and quantitative design (*n* = 1) [29]. All articles reported on nonexperimental studies. One article used a qualitative approach to analyze multiple individual HFR interventions [31], while others reported on one individual intervention each. One HFR intervention was studied in two separate articles, each using a different analytical approach and participant group [32, 36]. All studies reported on factors associated with both initial implementation and sustainment of HFR environment changes. Two studies had an overt focus on the sustainment of HFR environment changes [29, 32], while the 13 remaining articles [27, 28, 30, 31, 33, 34, 35, 36, 37, 38, 39, 40, 41] reported on these only briefly. Factors from the latter 13 studies were included in the synthesis if these were clearly reported as associated with sustainment.

**TABLE 2:** Characteristics of articles included in the review on determinants of sustained healthy food environment changes.

Author(s), year	Country; jurisdiction (if applicable)	Aim of study	Study design	Number and types of participating food outlets/settings	Individual participants	Number of individual participants	Initial implementation year; intervention length; follow‐up year
Adams et al., 2012 [37]	United Kingdom; England	To conduct a process evaluation of the convenience store intervention (“Change4Life”).	Case study, mixed methods	74 convenience stores	Store owners and/or managers, policymakers, “other professionals involved in intervention leadership and delivery”	10	2008; 8 months; 2011
Boelsen‐Robinson et al., 2020 [35]	United Kingdom; England	To identify how local governments can facilitate implementation and overcome barriers to healthy food service interventions, using the case study of the Healthier Catering Commitment, a voluntary initiative implemented in London.	Case study, qualitative	Independent restaurants/fast food outlets (number unclear)	Health promotion practitioners, policymakers, intervention staff, and other individuals “who could give context to the intervention”	22	2012 (policy); N/A (ongoing policy); 2016 (or later)
Boys et al., 2021 [38]	United States; North Carolina	To (1) explore factors influencing the implementation and participation in the “Healthy Food Small Retailer Program” from storeowners' perspectives and (2) evaluate the program's impact and effectiveness.	Multiple case study, mixed methods	Eight corner stores	Store owners and/or managers	7	2016; 24 months; 2019
Gittelsohn et al., 2014 [31]	United States; Maryland, Minnesota, North Carolina and Pennsylvania	To document implementation challenges and opportunities associated with small store interventions.	Multiple case study, qualitative	727 small stores/corner stores (from four case studies)	Store owners and/or managers, store staff, intervention staff	Unclear	2004–10; 12–24 months; 2011–12
Greaney et al., 2014 [34]	United States; Massachusetts	To examine barriers and facilitators to implementing and sustaining Healthy Choices, a 3‐year obesity prevention intervention in Massachusetts middle schools.	Case study, qualitative	10 schools (number of school food outlets unclear)	Canteen staff, school administrator, school coordinator, teachers	56	2004–06; 36 months; 2007–06
Law et al., 2021 [27]	Australia; Western Australia	To explore food retailers' understanding of a mandated food and nutrition policy, barriers and enablers to its implementation, policy impacts, and recommendations for ongoing compliance.	Case study, qualitative	44 food outlets in healthcare facilities	Store owners and/or managers	12	2008 (policy), 2018 (compliance target); N/A (ongoing policy); 2020
Levay et al., 2018 [28]	Canada; British Columbia	To investigate the processes associated with district‐level implementation of British Columbia's school food and beverage sales policy.	Multiple case study, qualitative	62 schools (number of school food outlets unclear)	Canteen managers and/or owners, school staff, district‐level staff, public health stakeholders	32 (interviews)	2005 (policy), 2014 (iteration); N/A (ongoing policy); Not reported (follow‐up 24 + months after initial implementation)
Lynch et al., 2023 [32]	Canada; Ontario	To describe the experiences and key factors affecting the feasibility and sustainability of a healthy corner store intervention, which was not sustained, with a specific focus on the fresh fruit and vegetables sales.	Case study, qualitative research	Eight corner stores/convenience stores	Store owners and/or managers	8	2017; 6 months; 2019
Lynch & Giles, 2024 [36]	Canada; Ontario	To explore real‐world barriers to implementing Ottawa's discontinued “Healthy Corner Store” program, using Diffusion of Innovations theory and insights from public health unit (PHU) staff and retailers.	Case study, qualitative research	Eight corner stores/convenience stores	Store owners and/or managers; health promotion practitioners	9 (6 retailers, 2 PHU staff)	2017; 6 months; 2019
McGuire et al., 2020 [30]	United States; Minnesota	To investigate small food store managers' attitudes toward government food retail regulations and assess whether their views shifted following the “2014 Minneapolis Staple Foods Ordinance,” which mandated stocking specific healthy products.	Case study, mixed methods	147 small stores (pharmacies, convenience stores, gas stations etc.)	Store owners and/or managers	147	2014 (policy), 2015 (implementation target); N/A (ongoing policy); 2017
Pettigrew et al., 2018 [29]	Australia; Western Australia	To evaluate the implementation and acceptance of a Healthy Food and Drink Policy for schools a decade after its introduction and its impact on canteen profitability.	Quantitative	79 schools (number of school food outlets unclear)	Canteen managers, school principals, teachers, presidents of parents, and citizen committees	307	2007 (policy); N/A (ongoing policy); 2016
Rosin et al., 2024 [41]	New Zealand	To explore hospital food providers' and public health dietitians' experiences with Policy implementation and identify tools and resources to support it.	Case study, qualitative	Seven food outlets in healthcare facilities	Store owners and/or managers and staff; health promotion practitioners; dietitians	12	2016 (policy), 2018 (implementation target); N/A (ongoing policy); 2022
Vargas et al., 2023 [40]	Australia; Victoria	To explore and understand how co‐creation was applied in a supermarket intervention (“Eat Well, Feel Good Ballarat” project).	Case study, qualitative	Grocery stores/supermarkets (number unclear)	Store owners and/or managers, stakeholders representing health services, government organizations, academic institutions, and community (roles not clearly defined)	Unclear	2020; Not reported; 2022
Wood et al., 2024 [39]	United States, California	To describe how a large county government incorporated healthy nutrition standards and practices into its food service contracting process.	Case study, mixed methods	15 county departments (number of food outlets unclear)	Policymakers; other county government staff	Interviews: 13 (not entirely clear) Survey: 34	2011; N/A (ongoing policy); 2012 (interviews), 2018 (surveys); 2021 (review of food vendor contracts)
Young et al., 2018 [33]	United States; Wisconsin	To explore the successes and challenges of a collaborative pilot project aimed at increasing healthy food availability in Milwaukee corner stores.	Case study, mixed methods	Five corner stores	Store owners and/or managers and staff	2–4 staff members in five stores	Not reported; 24 months; Not reported (follow‐up 16–24 months after initial implementation)

### 3.3 Quality Assessment

Quality assessment results are reported in the Additional file IV. Eight of the nine articles reporting qualitative studies rated “yes” for all related items [27, 28, 32, 34, 35, 40]. Two of the five mixed‐method articles received a “yes” rating for all related items [37, 38]. Common items rated as “no” or “unclear” for the mixed‐method studies were “Are the different components of the study effectively integrated to answer the research question?”, “Are the outputs of the integration of qualitative and quantitative components adequately interpreted?”, and “Are divergences and inconsistencies between quantitative and qualitative results adequately addressed?”. These issues often arose from limitations in reporting, where only the qualitative approach was detailed while the quantitative approach received minimal attention. The quantitative article received lower quality ratings due to overall reporting limitations [29].

### 3.4 Types of Settings and Participants

Eight articles reported studies conducted in grocery stores, small/convenience stores or similar settings [30, 31, 32, 33, 36, 37, 38, 40], three in for‐profit school canteens [28, 29, 34], two in healthcare settings [27, 41], one in food outlets in government settings [39], and one in independent restaurants/food service outlets [35]. Individual participants (interviewees and survey respondents) represented mainly food retailers or store owners, managers, outlet staff, and program staff or other people supporting the implementation of the intervention. Participants of school‐ and healthcare‐based evaluations also represented other people working in these settings (e.g., school principals, healthcare workers). The number of participants within the included articles ranged from seven to 307 and was unclear in two articles [31, 40].

### 3.5 Types of Interventions

Eight articles reported specific in‐store intervention programs [31, 32, 33, 34, 36, 37, 38, 40], while seven focused on policy interventions [27, 28, 29, 30, 35, 39, 41]. Thirteen articles reported interventions targeting the whole food outlet product offering (food and drinks) [27, 28, 29, 30, 31, 32, 34, 35, 36, 38, 39, 40, 41], and two studies [33, 37] focused only on fruit and vegetables. Almost all reported on interventions focused on increasing the availability of healthy food and/or drinks [27, 28, 29, 30, 31, 32, 33, 34, 35, 36, 37, 38, 39, 41] and seven reported on interventions focused on limiting the availability of less healthy food and/or drinks [27, 28, 29, 34, 35, 39, 41]. Nine articles reported interventions using promotional materials and/or activities [27, 29, 31, 33, 35, 36, 37, 38, 40], eight utilized product placement [27, 29, 31, 33, 35, 37, 40, 41], five involved consumer education [31, 33, 34, 39, 40], and two targeted product prices [31, 38].

### 3.6 Intervention Length and Follow‐Up Period

The intervention length varied, with three articles reporting on interventions running for less than 12 months [32, 36, 37], three articles reporting on interventions running for between 12 and 24 months [31, 33, 38], and one article reporting on an intervention running for 36 months [34]. Seven articles reported on policy interventions without a specified duration (i.e., ongoing policies) [27, 28, 29, 30, 35, 39, 41], and one article did not report the intervention length [40]. The number of years since the initial implementation of the intervention to follow‐up varied between 2 and 10 years, with most articles (*n* = 11) covering periods of 2 to 3 years, two covering a period of 4 years [35, 41], and two covering a period of 9 to 10 years [29, 39]. Only three articles reported on interventions that had been without external implementation support for more than 2 years [29, 32, 39].

### 3.7 Determinants Identified as Influencing the Sustainment of Healthy Changes in In‐Store Food Retail Environments

The most frequently identified determinants reported to influence the sustainment of HFR environment changes are summarized in Figure 2, along with the number of articles reporting each factor and domain. See Additional file VI for the full list of identified determinants and detailed findings and Additional file VII for the thematic analysis of sustainment‐related results.

**FIGURE 2:**
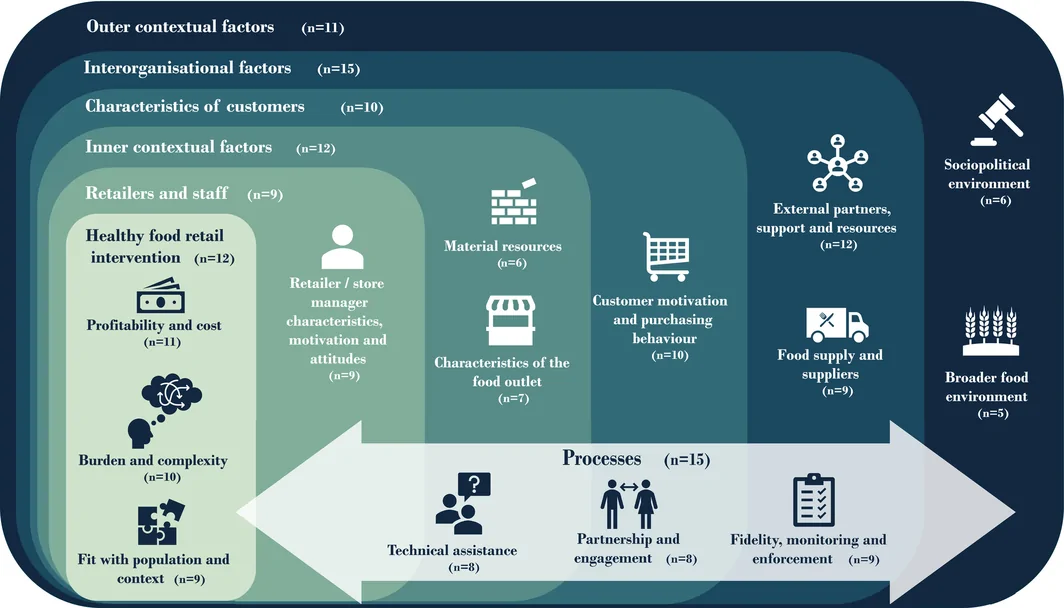
The most frequently identified domains and factors influencing the sustainment of healthy food retail environment changes, along with the number (*n*) of articles reporting them, organized according to the Integrated Sustainability Framework adapted for the food retail context.

All domains from the coding framework adapted to this review were covered in the analysis. Additionally, a new domain “interorganizational factors” was developed to capture the direct relationships between food outlets and other organizations (e.g., suppliers), distinguishing these from the more distant influences considered under “outer contextual factors” (e.g., the broader food environment). The most frequently reported domains were “processes” and “interorganizational factors” (*n* = 15 articles), followed by “characteristics of the intervention” (*n* = 12), “inner contextual factors” (*n* = 12), “outer contextual factors” (*n* = 11), “characteristics of customers” (*n* = 10), and “characteristics of retailer” (*n* = 9). Barriers and facilitators were not considered as separate determinants because many determinants can act as either (e.g., “external funding” can be a facilitator, and “lack of external funding” can be a barrier). Most determinants were related to sustainment of product availability changes rather than sustainment of other HFR strategies (e.g., placement and price). There was some evidence on the sustainment of promotional materials [32, 36, 37, 40], though findings were more limited in this area. There were no findings on the sustainment of pricing strategies.

#### 3.7.1 Outer Contextual Factors

Most articles (*n* = 11) [28, 29, 30, 31, 32, 34, 36, 38, 40, 41] reported at least one outer contextual factor influencing the sustainment of HFR changes; however, there was some variability in the factors identified across the studies. The most frequently identified “outer contextual factors” were “sociopolitical environment” (*n* = 6 articles) [28, 29, 30, 31, 34, 41], “broader food environment” (*n* = 5 articles) [30, 32, 36, 38, 41], and “external recognition of healthy food retail practice” (*n* = 4 articles) [31, 36, 38, 40]. For example, policies were often described as mechanisms to enforce healthy food practices among retailers but were not considered sufficient alone to facilitate sustainment of healthy changes in the settings they targeted (e.g., school canteens, small stores) [28, 30]. The broader food environment was considered to influence the sustainment of HFR changes primarily through the overall food system's limited support for healthier choices (e.g., limited availability of healthy foods from manufacturers, fluctuating prices of healthy foods) [30, 32, 41]. Media exposure [40] and certification programs [31] highlighting healthy changes in the food outlet product offerings were considered beneficial “external recognition of HFR practice” for sustained HFR changes.

#### 3.7.2 Interorganizational Factors

All articles identified “interorganizational” determinants related to the direct relationships between food retailers and other organizations potentially influencing their food offerings [27, 28, 29, 30, 31, 32, 33, 34, 35, 36, 37, 38, 39, 40, 41]. The most frequent factor was “external partners, support, and resources” (*n* = 12 articles) [27, 28, 29, 30, 31, 32, 33, 34, 35, 36, 38, 40]. Participants described that HFR practices were often not prioritized by food retailers and suggested that sustainment needs to be driven and supported by external partners whose primary focus is population health (e.g., universities, government health departments, and nongovernmental health promotion organizations). The second most frequent factor within this domain was “food supply and supplier related factors,” with nine studies reporting its importance for sustained food retail environment changes [27, 30, 31, 32, 33, 36, 38, 40, 41]. Limited availability of healthy alternatives from suppliers [27, 30, 32, 33, 38, 41] and higher costs associated with these alternatives [27, 32, 38] were identified as challenges. Additionally, current contractual obligations with suppliers were considered unsupportive to sustaining HFR changes [27, 32, 40].

#### 3.7.3 Characteristics of Customers

Eleven articles reported customer‐related factors influencing the sustainment of HFR changes with the most frequent one being “customer motivation and purchasing behavior” (*n* = 10 articles) [27, 31, 32, 34, 35, 36, 37, 38, 40, 41]. A key consensus was that if customers are not interested in the healthy food products, food retailers are unlikely to maintain these items in their product offerings. For example, some small store retailers in the included studies perceived that customers prefer to buy healthier options (e.g., fruit and vegetables) from other outlets offering a wider range and competitive prices (e.g., small store versus supermarket) [32, 37]. Food prices were especially important to customers on lower incomes, while retailers found it challenging to offer these options at lower prices due to the higher associated stocking costs [32, 35, 37]. Moreover, two articles focusing on small stores noted that customers are less likely to notice or try new healthy products unless these are actively promoted [31, 38]. Constant updates to promotional materials to avoid customers becoming “store‐blind” to these were considered essential for keeping customers interested in the healthy product offering in grocery and small stores [31, 40]. Some small store retailers also believed that making healthy products readily available and accessible would eventually increase customer purchases and their food outlet's ability to compete against nearby food outlets, motivating these retailers to sustain healthy changes over time [32, 37, 38].

#### 3.7.4 Inner Contextual Factors

Twelve articles reported inner contextual factors influencing the sustainment of HFR changes [28, 30, 31, 32, 34, 35, 36, 37, 38, 39, 40, 41], with the most frequently identified factor being “characteristics of the food outlet/retail organization” (*n* = 7 articles) [30, 31, 36, 38, 40, 41]. For example, participants from small and convenience stores reported the types of customers they attracted and their purchasing behavior (e.g., buying tobacco, newspapers, and snacks) as influenced by their outlet and neighborhood characteristics. These retailers did not consider certain healthy foods in the product offering (e.g., fruit and vegetables) relevant to their clientele, impacting their willingness and financial capability to maintain such changes. “Material resources” were another frequently reported factor (*n* = 6 articles) [31, 32, 35, 37, 41]. For example, limited resources to replace worn‐out or outdated promotional materials (e.g., posters highlighting healthy foods), or to fix broken storage or display equipment were reported as barriers to sustained HFR changes.

#### 3.7.5 Characteristics of the Retailer

Factors related to retailer characteristics were the least frequently identified (*n* = 9) [27, 28, 31, 32, 36, 37, 38, 40, 41]. Nine articles reported factors related to “retailer and/or store manager characteristics, motivation and attitudes.” Specific examples included a lack of motivation or incentive to maintain healthy changes, often driven by observing other food retailers not implementing similar changes, creating a sense of unfairness, and/or a lack of monitoring or enforcement [28, 37, 41]. Also, while food retail managers might be supportive of HFR interventions, this may not be sufficient to ensure the sustainment of such interventions due to competing outlet priorities and other ongoing initiatives [32, 36, 38, 40]. Some small and convenience store retailers demonstrated motivation to sustain healthy changes by actively promoting healthier products and introducing new healthy items in the product offering [32, 36, 37, 38], even when this was associated with an increased workload [38] or initial financial losses [37]. However, financial trade‐offs (e.g., higher cost of healthy food, costs related to food waste) still posed challenges to sustaining healthy changes in their product offerings [32, 36, 37, 38].

#### 3.7.6 Characteristics of the Intervention

Most articles (*n* = 12) identified at least one intervention‐related factor influencing the sustainment of HFR changes [27, 29, 30, 31, 32, 33, 34, 37, 38, 39, 40, 41]. “Profitability and cost” [27, 30, 31, 32, 34, 35, 36, 37, 38, 40, 41] (*n* = 11 studies) and “burden and complexity” [27, 30, 31, 32, 33, 36, 37, 38, 40, 41] (*n* = 10 studies) were the most frequently reported intervention‐related factors. Retailers faced significant financial and operational challenges in stocking and maintaining healthy changes in their product offerings due to issues such as spoilage and wastage, equipment failures, and the unpredictability of food pricing. More than half of the included articles reported that the maintenance of HFR changes demanded additional time and resources, including identifying healthy products, sourcing from suppliers, managing inventory, and handling food waste from perishable items [27, 30, 32, 33, 37, 38, 40]. “Fit with population and context” was also a frequently reported factor influencing the sustainment of HFR changes (*n* = 9 articles) [27, 30, 31, 32, 34, 35, 37, 39, 41]. Retailers reported tailoring interventions to the unique characteristics of different food outlets is important [30, 32, 37]. To consider the intervention's fit with the context, participants referred to factors influencing sustained changes relevant to other domains, such as food outlet characteristics (e.g., typical product offerings).

#### 3.7.7 Processes

All articles (*n* = 15) identified at least one determinant of sustained HFR changes relating to the “processes” domain [27, 28, 29, 30, 31, 32, 33, 34, 35, 36, 37, 38, 39, 40, 41], most frequently “fidelity, monitoring and enforcement” [28, 30, 31, 34, 35, 36, 37, 39, 41]. Ongoing monitoring or enforcement of HFR changes was considered essential for ensuring retailers maintain healthy practices [28, 30, 31, 34, 35, 36, 37, 39, 41], with some articles attributing unsuccessful sustainment efforts to a lack of monitoring [28, 30, 37]. Other frequently reported factors included “partnership and engagement” [29, 30, 31, 32, 34, 37, 40, 41] (*n* = 8) and “technical assistance, support, and supervision” [27, 31, 33, 36, 37, 38, 39, 40] (*n* = 8). For example, establishing and maintaining connections with health promotion agencies or universities were often considered essential [30, 31, 32, 33, 34, 38, 40]. Technical assistance (e.g., provision of practical and up‐to‐date resources) was often associated with the need to share responsibilities with multiple stakeholders, including other food outlets and health‐promoting organizations, to achieve sustained HFR changes [27, 31, 35, 40].

### 3.8 Implementation and Sustainment Support Offered

Almost all articles (*n* = 14) reported on some kind of support offered to facilitate the implementation and/or maintenance of HFR changes. The most commonly reported support strategies were related to concepts “use evaluative and iterative strategies (e.g., monitoring)” (*n* = 11 articles) [27, 29, 30, 31, 32, 33, 34, 35, 36, 39, 40] and “develop stakeholder interrelationships” (*n* = 10 articles) [27, 31, 32, 33, 34, 35, 36, 37, 38] Specific examples of these included compliance audits and regular follow‐ups [27, 29, 30, 31, 32, 33, 34, 35, 36, 39, 40] and practical implementation support provided by external partners [27, 31, 32, 33, 34, 35, 36]. Most reported support strategies targeted common determinants of sustained HFR changes (Table 3); however, certain strategies, such as initial staff training [31, 33, 34, 38] and external financial support [31, 33, 34, 37, 38], appeared to be less relevant to sustainment.

**TABLE 3:** Intervention support strategies reported in articles included in the systematic review on determinants of sustained healthy food retail changes, and their alignment with the most frequently reported (≥ 5 articles) determinants of sustained healthy food environment changes and Sustainment‐Explicit Expert Recommendations for Implementing Change (ERIC) glossary.

Most common sustainment determinants reported in articles^a^		Evidence‐based implementation and sustainment strategies^b^			Relevant intervention support strategies reported in articles
Domain	Factor	Category	Strategy types	Sustainment‐specific considerations	
Outer contextual factors	Sociopolitical environment	Change infrastructure (structural changes)	Mandate change	Prioritize both policy implementation and sustainment	Policy mandates [27, 28, 29, 30]
	Broader food environment		Mandate change (broader food environment)^c^ Change regulatory or fiscal policies (e.g., food manufacturing, taxation laws)^c^	—	—
Interorganizational factors	External partners, support and resources	Provide interactive assistance	Provide supervision	Provide ongoing support and supervision	External partners overseeing and supporting the implementation of HFR changes [27, 31, 32, 33, 34, 35, 36]
		Develop stakeholder interrelationships	Promote network weaving Build a coalition	Maintain networks and partnerships to support sustainment; sustainment should be a shared vision	
		Change infrastructure (structural changes)	Start a dissemination organization	This organization is responsible for supporting sustained changes	—
	Food supply and supplier related factors		Mandate change (suppliers)^c^ Change regulatory or fiscal policies (e.g., food manufacturing, taxation laws)^c^	—	—
	Competition	Engage consumers	Increase demand	—	Promoting healthy food retail changes to the community and/or consumer education [31, 35, 36, 37]
Characteristics of customers	Customer motivation and purchasing behavior	Engage consumers	Increase demand Prepare consumers to be active participants	—	Promoting healthy food retail changes to the community and/or consumer education [31, 35, 36, 37]
	Customer characteristics	Use evaluative and iterative strategies	Conduct local needs assessment Obtain and use consumer feedback	Conduct ongoing assessments to ensure changes remain aligned with consumer needs	—
Inner contextual factors	Material resources	Change infrastructure (structural changes)	Change physical structure and equipment	Regularly revise and update materials to ensure continued relevance and effectiveness	Provision of equipment (e.g., fresh food display) [31, 33, 38] Provision of materials (e.g., promotional materials, guidelines) [31, 32, 35, 36, 37]
		Train and educate stakeholders	Distribute educational materials		
	Characteristics of the food outlet (business)	Provide interactive assistance	Promote adaptability	Continue to assess and adapt the fit of the intervention	—
Characteristics of the retailer	Retailer and/or store manager characteristics, motivation and attitudes	Utilize financial strategies	Alter incentive structures	Ensure incentives address barriers to sustained changes	Access to new equipment (e.g., fresh food display) [31, 33, 38]
			Incorporate the intervention into contractual or funding obligations (e.g., requiring healthy changes)^c^	—	Integrate healthy nutrition standards and food retail practices into a local government's contracting process with food retailers [39]
		Change infrastructure (structural changes)	Change liability mechanisms to make retailers more willing to sustain the intervention^c^	—	
Characteristics of the intervention	Fit with population and context	Use evaluative and iterative strategies	Conduct local needs assessment Obtain and use consumer feedback	Conduct ongoing assessments to ensure changes remain aligned with retailer and consumer needs	Community and retailer consultation [30, 40]
		Develop stakeholder interrelationships	Capture (and share) local knowledge Conduct local consensus discussions		
		Provide interactive assistance	Promote adaptability	Continue to assess and adapt the fit of the intervention	—
	Profitability and cost	Engage consumers	Increase demand	—	Promoting healthy food retail changes to the community and/or consumer education [31, 35, 36, 37]
	Burden and complexity	Provide interactive assistance	Provide local technical assistance Facilitation	Technical assistance should address challenges specifically related to sustainment	Technical assistance from a health promotion practitioner or dietitian or intervention staff [27, 31, 32, 33, 34, 35, 36, 37, 38]
Processes	Fidelity, monitoring and enforcement	Use evaluative and iterative strategies	Audit and provide feedback Develop and organize quality monitoring systems Purposely reexamine the implementation	Continuous monitoring and communication to maintain changes over time	Compliance audits and regular follow‐ups during the study period [27, 29, 30, 31, 32, 33, 34, 35, 36, 39, 40]
		Support retail managers and staff	Remind retailers		
	Partnership and engagement	Develop stakeholder interrelationships	Promote network weaving Build a coalition	Maintain networks and partnerships to support sustainment; sustainment should be a shared vision	Connecting food outlets with key stakeholders [36, 37, 38]
	Technical assistance, support and supervision	Provide interactive assistance	Provide local technical assistance Facilitation	Technical assistance should address challenges specifically related to sustainment	Technical assistance from a health promotion practitioner or dietitian or intervention staff [27, 31, 32, 33, 34, 35, 36, 37, 38]
		Train and educate stakeholders	Provide ongoing consultation	Consultation should be continuous	
	Increasing or creating consumer demand for healthy options	Engage consumers	Increase demand Prepare consumers to be active participants	—	Promoting healthy food retail changes to the community and/or consumer education [31, 35, 36, 37]
	Communication	Support retail managers and staff	Remind retailers Provide data on intervention outcomes (e.g., profitability, population health)^c^	Continuous communication to maintain changes over time	Compliance audits and regular follow‐ups during the study period [27, 29, 30, 31, 32, 33, 34, 35, 36, 39, 40]
		Use evaluative and iterative strategies	Communicate with stakeholders the continued benefits of the intervention		—

^a^ Factors cited by 5 or more sources.

^b^ Sustainment‐Explicit Expert Recommendations for Implementing Change (ERIC) glossary [14].

^c^ ERIC strategy category or type adapted to food retail context.

## 4 Discussion

This review identified the determinants associated with sustained HFR environment changes aimed at improving the healthiness of consumer food purchases in consumer‐facing food retail settings. Determinants related to sustained HFR environment changes across all domains of the adapted ISF [20], as well as a new developed domain (“interorganizational factors”). The most frequently reported domain across all settings was “processes.” Factors mapped to this domain suggested the need for continuous oversight of external health‐promoting agencies, consumer and community engagement, and collaborative approaches to ensure the long‐term sustainment of HFR changes. Some of the most frequently reported factors were related to the “characteristics of the intervention” domain, suggesting that HFR changes need to be financially viable, manageable in time and effort required, and tailored to the specific needs of the food outlets and their customers. Most articles in the review focused on small stores or similar settings, with few studies examining the long‐term sustainment (at least 2 years) of HFR changes in other settings (e.g., supermarkets, school canteens, and food outlets in public settings). This makes it challenging to compare determinants across different setting types and highlights needs for more research in this area. Practical support and compliance audits by external partners were the most frequently reported forms of assistance offered to food outlets, addressing key determinants of sustained HFR change (“partnerships and engagement”; “fidelity, monitoring, and enforcement”; “technical assistance”). However, successful sustainment of HFR changes likely depends on the consistency and frequency of the support provided by external partners [31, 34, 37]. Moreover, none of the intervention support strategies reported in the included studies clearly addressed “food supply and supplier related factors,” which was one of the most frequently reported determinants of sustained changes.

Addressing multiple key determinants across different domains is essential for sustainment of HFR changes, given that all domains were identified in most of the included articles in this review. We found that HFR interventions are more likely to be successful when they are aligned across multiple socio‐ecological levels, aligning with an umbrella review by Gupta et al. [13]. For example, while formal or mandatory policies targeting food outlet practices are beneficial (“outer context”), they are often insufficient to sustain HFR changes on their own. This finding is consistent with a review of business outcomes of HFR interventions [10] and highlights the need for ongoing external support, oversight (“interorganizational factors”), and monitoring or enforcement (“processes”) of policy‐driven changes to ensure sustained changes. The finding that multiple domains were frequently reported in this review differs from Shoesmith et al.’s [18] findings that inner contextual factors are the key to sustaining health promotion interventions in schools and early learning settings. Educational settings may have a clearer organizational role in promoting the health of their “end‐user” population (children) compared with publicly accessible food retail settings, making the sustainment of interventions in educational settings more reliant on school organizational behavior. In contrast, HFR interventions may rely more on the behaviors of their end‐user population (consumers), as these organizations are profit‐driven and may have a less defined role in supporting population health.

Some of the most frequently identified determinants of sustainment across the current and Gupta et al.’s [13] umbrella review related to “profitability and cost” and “partnership and engagement.” While this overlap may result from shared articles between the reviews, similar findings have also been reported in more recent studies with shorter follow‐up periods (less than 2 years) or unclear follow‐up timelines, focusing on small stores in the United States [42], Australian supermarkets and grocery stores [43, 44], and food outlets in government‐owned settings [10, 45]. These consistent findings suggest that profitability and cost of intervention components, along with a collaborative approach, should be integral parts of sustainment strategies. For example, ensuring the financial viability of HFR environment changes by increasing consumer demand (e.g., promotional efforts), while engaging and sharing responsibilities with key stakeholders (e.g., customer feedback, health‐promoting agencies overseeing healthy changes) may support the sustainment of HFR changes in food outlets. Financial viability could also be supported at government level through external funding mechanisms (e.g., subsidies) or fiscal policy measures (e.g., taxes on less healthy items or ingredients) that may improve the affordability and competitiveness of healthier options.

### 4.1 Similarities and Differences Between Determinants of Implementation and Sustainment

As expected [14], several determinants of sustainment identified in our review overlapped with factors described as influencing the implementation phase of HFR interventions in previous reviews (e.g., Gupta et al. [13], Middel et al. [7]). These include the essential role of external partners, such as health promoting agencies overseeing implementation and holding food retailers accountable for sustainment of HFR changes. Additionally, profitability and costs related to HFR changes are important for both implementation and sustainment, though the strategies addressing these may differ. While financial support to retailers can facilitate the implementation of healthy changes [7], our review suggests that increasing demand for healthier options or lowering the wholesale prices of healthy items (and/or raising that of less healthy items) could better support sustained HFR changes.

The key difference in profitability‐ and cost‐related factors between implementation and sustainment phases may lie in managing “short‐term financial losses” and achieving “long‐term profitability.” Our findings suggest that achieving long‐term profitability by encouraging customers to purchase healthier options is likely essential for motivating retailers to sustain healthy changes over time. However, initial financial support may help retailers to overcome the fear of profit loss or offset potential short‐term losses that may arise when introducing healthier product offerings, losses that might otherwise lead retailers to quickly discontinue these changes [7, 36]. Factors that also seem more relevant to the implementation phase include retailer or staff skills and knowledge. We found that “characteristics of the intervention,” such as “burden, complexity, and intervention design,” were more frequently reported sustainment factors compared with “characteristics of the retailer.” By contrast, previous studies [7, 13, 35] have identified insufficient retailer or store manager knowledge and skills as key barriers to implementation. Ensuring that interventions are low‐burden, and easy to implement and sustain from the retailers' perspective may be more effective than focusing on upskilling retailers to sustain complex interventions [46]. Improving the accessibility of intervention components, such as using simple and straightforward criteria to identify healthy product options [27, 45], could facilitate the implementation and sustainment of healthy options in the product offering.

### 4.2 Practice and Research Implications

While this review identified key determinants of sustainment, aligning these with actionable sustainment strategies is also important. The findings suggest that multiple strategies addressing multi‐level determinants are required to support sustainment (refer to Table 3 for some examples). For example, jurisdictional mandatory HFR requirements [27, 28, 29, 30] could be strengthened by ensuring their real‐world adaptability and contextual fit [30], together with consistent auditing and monitoring systems [14]. However, evidence on the effectiveness of specific sustainment strategies in the HFR context remains limited, highlighting an important area for future research.

It is also important to note that sustainment should be considered alongside intervention effectiveness and value, as maintaining practices that do not produce meaningful population health benefits represents an inefficient use of resources. While evidence supports the effectiveness of HFR marketing strategies in influencing purchasing behaviors [1, 4, 5], variation across different retail contexts (e.g., supermarkets vs. foodservice) and long‐term impacts on dietary intake remain less understood, highlighting important areas for future research.

### 4.3 Strengths and Limitations of Included Articles

Most of the included articles (10 of 15) rated “yes” for all seven related items used in the MMAT [25], indicating high‐quality research designs and methodologies. However, a significant limitation was that most sustainment‐related findings were secondary outcomes of the study results, underscoring the need for more research explicitly focusing on the factors influencing sustained HFR changes. This finding aligns with Gupta et al. [13], highlighting the limited research on the determinants influencing the sustainment of HFR interventions. All studies included in the current review were conducted in high‐income countries, primarily in the United States, and focused mainly on small and convenience stores. This highlights the need for more research that includes a broader range of countries and settings. Moreover, many included studies evaluated existing interventions, likely primarily focusing on evaluating intervention components and processes, which may explain why our findings emphasize “processes” and “characteristics of the intervention” over “outer contextual factors.” Future research could more thoroughly identify the determinants of sustained HFR changes across various food retail contexts by exploring a broader range of “outer contextual factors” (e.g., the role of the food manufacturing industry in influencing the sustainment of food outlet practices [47]) and “inner contextual factors” (e.g., the impact of staff turnover on food outlet practices over time [10]). Lastly, the findings indicate that sustainment‐related terminology is not well‐established in food retail research. Many studies appear to use terms like “implementation” and “success” interchangeably with “sustainment,” “maintenance,” or “continuation,” making it challenging to distinguish determinants specific to sustained HFR changes from those related to adoption and implementation phases. We recommend clearly distinguishing adoption, implementation, and sustainment phases, using consistent terminology (e.g., implementation versus sustainment or maintenance). Sustainment should be clearly defined (e.g., as a stage “following implementation” or “after the withdrawal of initial implementation support”) [20], and sustainment strategies reported separately from implementation strategies, noting any overlap to enhance clarity and comparability [14].

### 4.4 Methodological Strengths and Limitations of the Review

This review is the first to apply an empirical, sustainment‐specific framework to investigate the key determinants of sustained changes in the food retail context. One of the main strengths of this review is its inclusion of various types of food outlets, providing a broad perspective on the multiple factors influencing sustainment. The focus on studies with a follow‐up period of more than 2 years after the initial implementation ensured that the identified determinants are based on participants' real‐world experiences rather than prospective perceptions. The restriction to studies published in English and those that did not meet the time criterion of a follow‐up period longer than 2 years may have excluded relevant research. While this review captures evidence on the determinants of sustainment as described by study participants, it does not necessarily confirm the effectiveness of the intervention or capture actual fidelity to intervention components over time. Lastly, the review did not specifically measure the follow‐up period after external support had ended, due to the limited number of studies reporting on this criterion, which may have impacted the comprehensiveness of the sustainment analysis.

## 5 Conclusions

This review provides a comprehensive synthesis of the factors influencing the sustainment of HFR changes across various food retail contexts. Our findings indicate that sustainment is influenced by a diverse range of determinants, with those related to “processes” being the most frequently reported. Factors within this domain highlight the importance of continuous oversight, active consumer and community engagement, and a collaborative approach to ensure the ongoing success of HFR interventions. While current intervention support strategies address many determinants of sustained changes, consistent and continuous support is likely to be needed for achieving long‐term sustainment. The findings from this review can guide future intervention development, the planning of long‐term healthy changes in food outlets, and the testing of sustainment strategies. However, evidence in this area remains limited, with most research focusing on the implementation of HFR changes, highlighting the need for future research to advance a comprehensive understanding of sustainment determinants and strategies. This evidence is essential for enabling long‐term HFR transformation across diverse settings and contexts, supporting consumers in making healthier food choices and contributing to long‐term population health improvements.

## Funding

BB and NI were supported by the Deakin University Postgraduate Research Scholarships (DUPRS). CNHM was funded through ZonMW Vidi project “Profits or public health?” (grant no. 09150172210046). AG is supported by the Victorian Health Promotion Foundation (VicHealth) fellowship. MRB is the recipient of an Australian Research Council Discovery Early Career Researcher Award (project number DE240100616) funded by the Australian Government. AG, BB, MB, and SY are researchers in a Centre of Research Excellence focused on retail food environments, funded by the Australian National Health and Medical Research Council (NHMRC) (GNT1152968 and GNT2024716). VB is a researcher within the NHMRC funded Centre of Research Excellence in Translating the Early Prevention of Obesity in Childhood (GNT2006999). SY is supported by an Australian Heart Foundation Future Leader Fellowship (106654). The opinions, analysis, and conclusions in this paper are those of the authors and should not be attributed to the Australian Government, the NHMRC or Australian Research Council.

## Conflicts of Interest

The authors declare no conflicts of interest.

## Supporting information


**DATA S1:** PRISMA checklists.
**DATA S2:** Full search strategies for each database.
**DATA S3:** Systematic review—screening checklist.
**DATA S4:** Critical appraisal tool and ratings.
**DATA S6:** Factors identified to influence the sustainment of healthy food retail (HFR) environment changes, organized by Integrated Sustainability Framework domains and factors.

**DATA S5:** Data extracted from the studies included in the systematic review.

## Data Availability

The data that supports the findings of this study are available in the supplementary material of this article.
