# Supplementary material for: The Determinants of Long‐Term Sustainment of Healthy Food Retail Environment Changes: A Systematic Review
Source: Obes Rev. 2026 Apr 28;27(11):e70143. doi: 10.1111/obr.70143 (PMC13655977; doi:10.1111/obr.70143)
Supplement: Supplementary file 1 [file OBR-27-e70143-s001.pdf]

Supplementary material

The determinants of long-term sustainment of healthy food retail environment changes: a systematic review

Bettina Backman*[1], Cédric NH Middel[2,3,4], Serene Yoong[1,5], Adyya Gupta[1], Noor Imad[1,6], Vicki Brown[1,7], Miranda R Blake[1]

[1] Deakin University, Geelong, Australia, Institute for Health Transformation, Global Centre for Preventive Health and Nutrition, School of Health and Social Development, Faculty of Health

[2] Amsterdam UMC, Department of Epidemiology & Data Science, Amsterdam, The Netherlands

[3] Amsterdam Public Health research institute, Amsterdam, The Netherlands

[4] Upstream Team, www.upstreamteam.nl, Amsterdam, The Netherlands

[5] College of Health, Medicine and Wellbeing, University of Newcastle, NSW Callaghan

[6] Swinburne University, Department of Nursing and Allied Health, School of Health Sciences

[7] Deakin University, Geelong, Australia, Deakin Health Economics (DHE), Institute for Health Transformation

*Corresponding author: Bettina Backman, bettina.backman@deakin.edu.au

Additional file I: PRISMA checklists

Additional file II: Full search strategies for each database

Additional file III: Systematic review - screening checklist

Additional file IV: Critical appraisal tool and ratings

Additional file V: Data extracted from the studies included in the systematic review

Additional file VI: Factors identified to influence the sustainment of healthy food retail (HFR) environment changes, organised by Integrated Sustainability Framework domains and factors

PRISMA 2020 Checklist

Additional file I: PRISMA checklists 2020

Section and Topic	Item #	Checklist item	Location where item is reported
**TITLE**			
Title	1	Identify the report as a systematic review.	p.1
**ABSTRACT**			
Abstract	2	See the PRISMA 2020 for Abstracts checklist.	PRISMA 2020 for Abstracts checklist
**INTRODUCTION**			
Rationale	3	Describe the rationale for the review in the context of existing knowledge.	Background p.2-3
Objectives	4	Provide an explicit statement of the objective(s) or question(s) the review addresses.	Background p.2-3
**METHODS**			
Eligibility criteria	5	Specify the inclusion and exclusion criteria for the review and how studies were grouped for the syntheses.	Methods, p. 4-5; Table 1
Information sources	6	Specify all databases, registers, websites, organisations, reference lists and other sources searched or consulted to identify studies. Specify the date when each source was last searched or consulted.	Methods, p. 5; Additional file II
Search strategy	7	Present the full search strategies for all databases, registers and websites, including any filters and limits used.	Additional file II
Selection process	8	Specify the methods used to decide whether a study met the inclusion criteria of the review, including how many reviewers screened each record and each report retrieved, whether they worked independently, and if applicable, details of automation tools used in the process.	Methods, p. 5; Additional file III
Data collection process	9	Specify the methods used to collect data from reports, including how many reviewers collected data from each report, whether they worked independently, any processes for obtaining or confirming data from study investigators, and if applicable, details of automation tools used in the process.	Methods, p. 6
Data items	10a	List and define all outcomes for which data were sought. Specify whether all results that were compatible with each outcome domain in each study were sought (e.g. for all measures, time points, analyses), and if not, the methods used to decide which results to collect.	Methods, p. 6-7;

Section and Topic	Item #	Checklist item	Location where item is reported
			Additional file V
	10b	List and define all other variables for which data were sought (e.g. participant and intervention characteristics, funding sources). Describe any assumptions made about any missing or unclear information.	Methods, p. 6-7; Additional file V
Study risk of bias assessment	11	Specify the methods used to assess risk of bias in the included studies, including details of the tool(s) used, how many reviewers assessed each study and whether they worked independently, and if applicable, details of automation tools used in the process.	Methods, p. 6
Effect measures	12	Specify for each outcome the effect measure(s) (e.g. risk ratio, mean difference) used in the synthesis or presentation of results.	n/a (thematic synthesis and frequency count used)
Synthesis methods	13a	Describe the processes used to decide which studies were eligible for each synthesis (e.g. tabulating the study intervention characteristics and comparing against the planned groups for each synthesis (item #5)).	Methods, p. 6-7
	13b	Describe any methods required to prepare the data for presentation or synthesis, such as handling of missing summary statistics, or data conversions.	Methods, p. 6-7
	13c	Describe any methods used to tabulate or visually display results of individual studies and syntheses.	n/a (thematic synthesis and frequency count used)
	13d	Describe any methods used to synthesize results and provide a rationale for the choice(s). If meta-analysis was performed, describe the model(s), method(s) to identify the presence and extent of statistical heterogeneity, and software package(s) used.	Methods, p. 6-7
	13e	Describe any methods used to explore possible causes of heterogeneity among study results (e.g. subgroup analysis, meta-regression).	n/a (thematic synthesis and frequency count used)
	13f	Describe any sensitivity analyses conducted to assess robustness of the synthesized results.	n/a
Reporting bias assessment	14	Describe any methods used to assess risk of bias due to missing results in a synthesis (arising from reporting biases).	n/a
Certainty	15	Describe any methods used to assess certainty (or confidence) in the body of evidence for an outcome.	n/a

Section and Topic	Item #	Checklist item	Location where item is reported
assessment			
**RESULTS**			
Study selection	16a	Describe the results of the search and selection process, from the number of records identified in the search to the number of studies included in the review, ideally using a flow diagram.	Results, p. 8; Figure 1
	16b	Cite studies that might appear to meet the inclusion criteria, but which were excluded, and explain why they were excluded.	Discussion, p. 22
Study characteristics	17	Cite each included study and present its characteristics.	Results, Table 2
Risk of bias in studies	18	Present assessments of risk of bias for each included study.	Results, p.9; Additional file IV
Results of individual studies	19	For all outcomes, present, for each study: (a) summary statistics for each group (where appropriate) and (b) an effect estimate and its precision (e.g. confidence/credible interval), ideally using structured tables or plots.	n/a
Results of syntheses	20a	For each synthesis, briefly summarise the characteristics and risk of bias among contributing studies.	Results p. 8-13, Table 2; Additional file IV
	20b	Present results of all statistical syntheses conducted. If meta-analysis was done, present for each the summary estimate and its precision (e.g. confidence/credible interval) and measures of statistical heterogeneity. If comparing groups, describe the direction of the effect.	Results (thematic synthesis) p. 13-17; Additional files VI & VII; Figure 2; Table 3
	20c	Present results of all investigations of possible causes of heterogeneity among study results.	n/a
	20d	Present results of all sensitivity analyses conducted to assess the robustness of the synthesized results.	n/a
Reporting biases	21	Present assessments of risk of bias due to missing results (arising from reporting biases) for each synthesis assessed.	n/a
Certainty of evidence	22	Present assessments of certainty (or confidence) in the body of evidence for each outcome assessed.	n/a
**DISCUSSION**			
Discussion	23a	Provide a general interpretation of the results in the context of other evidence.	Discussion p. 21-23

Section and Topic	Item #	Checklist item	Location where item is reported
	23b	Discuss any limitations of the evidence included in the review.	Discussion p. 23-24
	23c	Discuss any limitations of the review processes used.	Discussion p. 24
	23d	Discuss implications of the results for practice, policy, and future research.	Discussion p. 23-24
**OTHER INFORMATION**			
Registration and protocol	24a	Provide registration information for the review, including register name and registration number, or state that the review was not registered.	Methods p.4
	24b	Indicate where the review protocol can be accessed, or state that a protocol was not prepared.	Methods p.4, References
	24c	Describe and explain any amendments to information provided at registration or in the protocol.	n/a
Support	25	Describe sources of financial or non-financial support for the review, and the role of the funders or sponsors in the review.	Cover page
Competing interests	26	Declare any competing interests of review authors.	Cover page
Availability of data, code and other materials	27	Report which of the following are publicly available and where they can be found: template data collection forms; data extracted from included studies; data used for all analyses; analytic code; any other materials used in the review.	Cover page

From: Page MJ, McKenzie JE, Bossuyt PM, Boutron I, Hoffmann TC, Mulrow CD, et al. The PRISMA 2020 statement: an updated guideline for reporting systematic reviews. BMJ 2021;372:n71. doi: 10.1136/bmj.n71

For more information, visit: http://www.prisma-statement.org/

PRISMA 2020 Checklist

PRISMA 2020 for Abstracts Checklist

Section and Topic	Item #	Checklist item	Reported (Yes/No)
**TITLE**			
Title	1	Identify the report as a systematic review.	Yes
**BACKGROUND**			
Objectives	2	Provide an explicit statement of the main objective(s) or question(s) the review addresses.	Yes
**METHODS**			
Eligibility criteria	3	Specify the inclusion and exclusion criteria for the review.	Yes
Information sources	4	Specify the information sources (e.g. databases, registers) used to identify studies and the date when each was last searched.	Yes
Risk of bias	5	Specify the methods used to assess risk of bias in the included studies.	Yes
Synthesis of results	6	Specify the methods used to present and synthesise results.	Yes
**RESULTS**			
Included studies	7	Give the total number of included studies and participants and summarise relevant characteristics of studies.	Yes
Synthesis of results	8	Present results for main outcomes, preferably indicating the number of included studies and participants for each. If meta-analysis was done, report the summary estimate and confidence/credible interval. If comparing groups, indicate the direction of the effect (i.e. which group is favoured).	Yes
**DISCUSSION**			
Limitations of evidence	9	Provide a brief summary of the limitations of the evidence included in the review (e.g. study risk of bias, inconsistency and imprecision).	Yes
Interpretation	10	Provide a general interpretation of the results and important implications.	Yes
**OTHER**			
Funding	11	Specify the primary source of funding for the review.	No (reported on the cover page)
Registration	12	Provide the register name and registration number.	No (reported in methods)

From: Page MJ, McKenzie JE, Bossuyt PM, Boutron I, Hoffmann TC, Mulrow CD, et al. The PRISMA 2020 statement: an updated guideline for reporting systematic reviews. BMJ 2021;372:n71. doi: 10.1136/bmj.n71. This work is licensed under CC BY 4.0. To view a copy of this license, visit https://creativecommons.org/licenses/by/4.0/

Additional file II: Full search strategies for each database

**Database(s)**	**Hedge terms**				
	"Food retail outlet"	"Food products"	"Healthy food retail intervention"	"Determinants of sustainment"	*Other search criteria*
Central	("food retail" OR supermarket* OR Grocer* OR "convenience store" OR "small store" OR "corner store" OR "corner shop" OR "food outlet" OR "food service" OR Café OR "coffee shop" OR Kiosk OR Canteen OR "food provider" OR takeaway OR "take away" OR "takeout" OR "food delivery" OR "food environment"):ti,ab,kw<br><br>OR MeSH descriptor: [Supermarkets] explode all trees<br><br>OR MeSH descriptor: [Food Services] explode all trees	(health OR "nutritious" OR "nutritional quality" OR Unhealth* OR Discretionary OR Obesogenic OR Food* OR Drink* OR "Product offering" OR Beverage* OR Meal* OR Menu OR Lunch OR Snack* OR Fruit OR Vegetable* OR confectionery):ti,ab,kw<br><br>OR MeSH descriptor: [Diet, Healthy] explode all trees<br><br>OR MeSH descriptor: [Food] explode all trees	(Initiative* OR Intervention* OR Strateg* OR Polic* OR program* OR project* OR campaign* OR trial* OR regulation OR Offer* OR select* OR supply OR Availab* OR Pric* OR Plac* OR Promot* OR Marketing OR Access* OR Nudg* OR "Choice architecture" OR "best practice" OR discount*):ti,ab,kw<br><br>OR MeSH descriptor: [Marketing] explode all trees	AND (Determinant* OR Factor* OR Motivat* OR Barrier* OR Attribut* OR Facilitat* OR Challenge* OR Opportunit* OR Obstacle* OR Enabl* OR (impede or impediment) OR (hindrance or hinder) OR hurdle* OR correlate* OR mediat* OR contributor* OR associat* OR modif* OR confound* OR pattern* OR relation* OR predict*)<br><br>AND (Scal* OR Maint* OR Sustain* OR Change* OR Adopt* OR Long-term or "long term" OR Permanent OR Continu* OR "over time" OR Longitudinal OR Lasting OR Follow-up or "follow up" OR implement* OR complian* OR adherence OR routinization OR discontin* OR deadopt* or "de-adopt" OR durabil* OR institutionalization OR "capacity building" OR "knowledge utilization" OR (reaim or "re aim") OR epis OR "new normal"):ti,ab,kw<br><br>OR MeSH descriptor: [Program Evaluation] explode all trees	NOT TI (review or protocol)

Embase	(“food retail” OR supermarket* OR Grocer* OR “convenience store” OR “small store” OR “corner store” OR "corner shop" OR “food outlet” OR “food service” OR Café OR "coffee shop" OR Kiosk OR Canteen OR "food provider" OR takeaway OR “take away” OR “takeout” OR “food delivery” OR "food environment"):ti,ab,kw<br><br>OR ‘food service’/exp OR 'grocery store'/exp	(health OR “nutritious” OR “nutritional quality” OR Unhealth* OR Discretionary OR Obesogenic OR Food* OR Drink* OR “Product offering” OR Beverage* OR Meal* OR Menu OR Lunch OR Snack* OR Fruit OR Vegetable* OR confectionery):ti,ab,kw<br><br>OR ‘healthy diet’/exp	(Initiative* OR Intervention* OR Strateg* OR Polic* OR program* OR project* OR campaign* OR trial* OR regulation OR Offer* OR select* OR supply OR Availab* OR Pric* OR Plac* OR Promot* OR Marketing OR Access* OR Nudg* OR “Choice architecture” OR “best practice” OR discount*):ti,ab,kw	AND (Determinant* OR Factor* OR Motivat* OR Barrier* OR Attribut* OR Facilitat* OR Challenge* OR Opportunit* OR Obstacle* OR Enabl* OR (impede or impediment) OR (hindrance or hinder) OR hurdle* OR correlate* OR mediat* OR contributor* OR associat* OR modif* OR confound* OR pattern* OR relation* OR predict*)<br><br>AND (Scal* OR Maint* OR Sustain* OR Change* OR Adopt* OR Long-term or "long term" OR Permanent OR Continu* OR “over time” OR Longitudinal OR Lasting OR Follow-up or “follow up” OR implement* OR complian* OR adherence OR routinization OR discontin* OR deadopt* or "de-adopt" OR durabil* OR institutionalization OR "capacity building" OR "knowledge utilization" OR (reaim or "re aim") OR epis OR "new normal"):ti,ab,kw	NOT TI (review or protocol)
Global Health	AB/TI (“food retail*” OR supermarket* OR Grocer* OR “convenience store*” OR “small store*” OR “corner store*” OR "corner shop*" OR “food outlet*” OR “food service*” OR Café* OR "coffee shop*" OR Kiosk* OR Canteen* OR "food provider*" OR takeaway OR “take away” OR	AB/TI (“health*” OR “nutritious” OR “nutritional quality” OR Unhealth* OR Discretionary OR Obesogenic OR Food* OR Drink* OR “Product offering” OR Beverage* OR Meal* OR Menu OR Lunch OR Snack* OR Fruit OR Vegetable* OR confectionery)	AB/TI (Initiative* OR Intervention* OR Strateg* OR Polic* OR program* OR project* OR campaign* OR trial* OR regulation AND (Offer* OR select* OR supply OR Availab* OR Pric* OR Plac* OR Promot* OR Marketing OR Access* OR Nudg* OR “Choice	AND TX (Determinant* OR Factor* OR Motivat* OR Barrier* OR Attribut* OR Facilitat* OR Challenge* OR Opportunit* OR Obstacle* OR Enabl* OR (impede* or impediment*) OR (hindrance* or hinder*) OR hurdle* OR correlate* OR mediat* OR contributor* OR associat* OR modif* OR confound* OR pattern* OR relation* OR predict*)	NOT TI (review or protocol)

	“takeout” OR “food delivery” OR "food environment*")<br><br>OR DE "salad bars" OR OR DE "food stores" OR DE "restaurants" OR DE "fast food restaurants" OR DE "takeout restaurants" OR DE "cafes" OR DE "cafeterias"	OR DE "food" OR DE "breakfast" OR DE "dinner" OR DE "home delivered meals" OR DE "lunch" OR DE "school meals" OR DE "snacks" OR DE "meals" OR DE "confectionery" OR DE "candy" OR DE "lollies" OR DE "fast foods" OR DE "soft drinks" OR "beverages” OR DE "vegetables" OR DE "fruit"	architecture” OR “best practice” OR discount*))<br><br>OR DE "food marketing" OR DE "health promotion" OR DE "nutrition policy" OR DE "nutrition programmes"	AND AB/TI (Scal* OR Maint* OR Sustain* OR Change* OR Adopt* OR Long-term or "long term" OR Permanent OR Continu* OR “over time” OR Longitudinal OR Lasting OR Follow-up or “follow up” OR implement* OR complian* OR adherence OR routini?ation OR discontin* OR (deadopt* or "de-adopt*") OR durabil* OR institutionali?ation OR "capacity building" OR "knowledge utili?ation" OR (reaim or "re aim") OR epis OR "new normal" OR evaluation)<br><br>NOT AB "environmental* sustainab*"	
MEDLINE complete, CINAHL	AB/TI (“food retail*” OR supermarket* OR Grocer* OR “convenience store*” OR “small store*” OR “corner store*” OR "corner shop*" OR “food outlet*” OR “food service*” OR Café* OR "coffee shop*" OR Kiosk* OR Canteen* OR "food provider*" OR takeaway OR “take away” OR “takeout” OR “food delivery”)<br><br>OR (MM "Food Services+") OR (MM "Restaurants") OR (MM	AB/TI (“health*” OR “nutritious” OR “nutritional quality” OR Unhealth* OR Discretionary OR Obesogenic OR Food* OR Drink* OR “Product offering” OR Beverage* OR Meal* OR Menu OR Lunch OR Snack* OR Fruit OR Vegetable*)<br><br>OR (MH "Diet, Healthy") OR (MH "Food+") OR (MH "Fast Foods") OR (MH "Food and Beverages+") OR (MH "Food Ingredients+") OR (MH "Snacks") OR (MH "Nutritive Value+") OR (MH "Meals+")	AB/TI (Initiative* OR Intervention* OR Strateg* OR Polic* OR program* OR project* OR campaign* OR trial* OR regulation OR Offer* OR select* OR supply OR Availab* OR Pric* OR Plac* OR Promot* OR Marketing OR Access* OR Nudg* OR “Choice architecture” OR “best practice”)<br><br>OR (MM "Access to Healthy Foods") OR (MH "Health	TX (Determinant* OR Factor* OR Motivat* OR Barrier* OR Attribut* OR Facilitat* OR Challenge* OR Opportunit* OR Obstacle* OR Enabl* OR (impede* or impediment*) OR (hindrance* or hinder*) OR hurdle* OR correlate* OR mediator* OR contributor* OR association* OR modifier* OR confounder* OR pattern* OR relation* OR predictor*)<br><br>AND AB/TI (Scal* OR Maint* OR Sustain* OR Change* OR Adopt* OR Long-term or "long term" OR Permanent OR Continu* OR “over time” OR Longitudinal OR Lasting OR Follow-up or “follow up” OR complian* OR adherence OR routini?ation OR discontin* OR	NOT TI (review or protocol)

	"Supermarkets") OR (MM "Hospital Shops")	OR (MH "Breakfast") OR (MH "Lunch") OR (MH "Salads") OR (MH "Fruit+") OR (MH "Vegetables+") OR (MH "Sugar-Sweetened Beverages") OR (MH "Candy+")	Promotion+") OR (MH "Nutrition Policy+")	(deadopt* or "de-adopt*") OR durabil* OR institutionali?ation OR capacity building OR knowledge utili?ation OR (reaim or "re aim") OR "new normal" OR "post-implementation"<br><br>NOT AB ("environmental* sustainab*")<br><br>OR (MM "Change Management") OR (MH "Program Evaluation+") OR (MH "Follow-Up Studies") OR (MH "Guideline Adherence") OR (MH "Longitudinal Studies+"))	
Scopus	TITLE-ABS-KEY ("food retail" OR supermarket* OR grocer* OR "convenience store*" OR "small store*" OR "corner store*" OR "food outlet*" OR "food service*" OR café* OR kiosk* OR "coffee shop*" OR canteen* OR takeaway OR "take away" OR takeout OR "food delivery" OR "food environment" OR "nutrition environment" OR "retail environment")	AND TITLE-ABS-KEY (healthy OR unhealthy OR nutritious OR "nutritional quality" OR discretionary OR obesogenic) AND TITLE-ABS-KEY (food* OR drink* OR "product offering" OR beverage* OR meal* OR menu OR lunch OR snack* OR fruit OR vegetable* OR confectionery*)	AND TITLE-ABS-KEY (initiative* OR intervention* OR strateg* OR polic* OR program* OR project* OR campaign* OR trial* OR regulation) AND TITLE-ABS-KEY (offer* OR availab* OR pric* OR discount* OR plac* OR promot* OR marketing OR access* OR nudg* OR "choice architecture" OR "best practice")	AND ALL (determinant* OR factor* OR motivat* OR barrier* OR attribut* OR facilitat* OR challenge* OR opportunit* OR obstacle* OR enabl* OR impede* OR impediment OR hindrance* OR hurdle* OR correlate* OR mediator* OR contributor* OR association* OR modifier* OR confounder* OR pattern* OR relation* OR predictor*)<br><br>AND TITLE-ABS-KEY (scal* OR maint* OR sustain* OR change* OR adopt* OR "long-term" OR "long term" OR permanent OR continu* OR "over time" OR longitudinal OR lasting OR follow-up OR "follow up" OR complian* OR adherence OR routini?ation OR discontin* OR deadopt* OR "de-adopt*" OR durabil* OR institutionali?ation OR capacity building OR knowledge utili?ation OR (reaim OR "re aim") OR	AND NOT TITLE-ABS-KEY (review OR protocol)

				EPIS OR implement* OR "new normal" OR evaluation)	
Google scholar	(“food outlet” OR store OR supermarket OR canteen)	AND ("healthy food" or "food policy")		AND (sustain OR maintain OR continue OR discontinue OR “long term” OR "over time")	

Additional file III: Systematic review- screening checklist

Sustainment of Healthy Food Retail Environment Change – systematic review
Screening checklist

Abstract screening:

Q1. Is the paper a study protocol, editorial, systematic review, umbrella review, scoping review or other systematic-review-like study?

Yes -> exclude (or if relevant papers for discussion, tag with 'background/discussion')
No - continue to Q2

Q2. Does the study report on a particular HFR intervention (e.g., policy implementation; researcher-led intervention, etc.)?

Yes -> continue to Q3
Not sure -> save for full-text screening
No - exclude

Q3. Does the intervention target consumer-facing food retail or service outlets (where consumers make food purchases)?

Yes -> continue to Q4
Not sure -> save for full-text screening
No - exclude

Q4. Does the intervention target charitable organisations (e.g., food banks) or food service embedded in other services (e.g., hospital care)?

Yes -> exclude
Not sure -> save for full-text screening
No - continue to Q5

Q5. Does the intervention include changes in the in-store food environment that relate to at least one of the '4Ps of Marketing' strategies (Product availability, Placement, Price and Promotion)? *For example, menu changes relate to product availability*

Yes -> continue to Q6
Not sure -> save for full-text screening
No - exclude

Q6. Does the paper report on data that were collected at least 2 years (24 months) after the <u>initial</u> implementation?

Yes -> include
Not sure -> save for full-text screening
No -> continue to Q7

Q7. Does the paper report on data that were collected between 12-23 months after the initial implementation?

Yes -> add to 'maybe' folder
Not sure -> save for full-text screening
No -> exclude

Full-text screening:
Complete any questions that you answered 'not sure' during the abstract screening.

Full-Q1. Does the paper identify factors (barrier or facilitator or both) related to sustainment/maintenance of the in-store food environment changes (product availability, placement, price and promotion)?

Yes -> continue to Full-Q2
Not sure -> continue to Full-Q3
No -> continue to Full-Q3

Full-Q2. Are these factors identified in the results?

Yes -> include
No (e.g., only mentioned in the background) -> exclude

Additional file IV: Critical appraisal tool and ratings

Article	Screening questions		1. Qualitative studies					4. Quantitative descriptive studies					5. Mixed-method studies				
	Are there clear research questions?	Do the collected data allow to address the research questions?	1.1. Is the qualitative approach appropriate to answer the research question?	1.2. Are the qualitative data collection methods adequate to address the research question?	1.3. Are the findings adequately derived from the data?	1.4. Is the interpretation of results sufficiently substantiated by data?	1.5. Is there coherence between qualitative data sources, collection, analysis and interpretation?	4.1. Is the sampling strategy relevant to address the research question?	4.2. Is the sample representative of the target population?	4.3. Are the measurements appropriate?	4.4. Is the risk of nonresponse bias low?	4.5. Is the statistical analysis appropriate to answer the research question?	5.1. Is there an adequate rationale for using a mixed methods design to address the research question?	5.2. Are the different components of the study effectively integrated to answer the research question?	5.3. Are the outputs of the integration of qualitative and quantitative components adequately interpreted?	5.4. Are divergences and inconsistencies between quantitative and qualitative results adequately addressed?	5.5. Do the different components of the study adhere to the quality criteria of each tradition of the methods involved?
**Quantitative studies**																	
Pettigrew 2018	Yes	Can't tell						Yes	Can't tell	Can't tell	No	Yes					
**Qualitative studies**																	
Lynch 2024	Yes	Yes	Yes	Yes	Yes	Yes	Yes										
Lynch 2021	Yes	Yes	Yes	Yes	Yes	Yes	Yes										
Boelsen-Robinson 2021	Yes	Yes	Yes	Yes	Yes	Yes	Yes										
Greaney 2014	Yes	Yes	Yes	Yes	Yes	Yes	Yes										
Law 2021	Yes	Yes	Yes	Yes	Yes	Yes	Yes										
Levay 2019	Yes	Yes	Yes	Yes	Yes	Yes	Yes										
Vargas 2023	Yes	Yes	Yes	Yes	Yes	Yes	Yes										
Gittelsohn 2014	Yes	Yes	Yes	Yes	Yes	No	Yes										
Rosin 2024	Yes	Yes	Yes	Yes	Yes	Yes	Yes										
**Mixed-method studies**																	
Wood 2025	Yes	Yes	Yes	Yes	Yes	Yes	Yes	*Can't tell	Yes	Yes	*No	*Can't tell	Yes	Yes	Yes	Yes	No*
McGuire 2021	Yes	Yes	Yes	Yes	Yes	Yes	Yes	Yes	Yes	Yes	Yes	Yes	No	No	No	No	Yes
Adams 2012	Yes	Yes	Yes	Yes	Yes	Yes	Yes	Yes	Yes	Yes	Yes	Yes	Yes	Yes	Yes	Yes	Yes
Boys 2021	Yes	Yes	Yes	Yes	Yes	Yes	Yes	Yes	Yes	Yes	Yes	Yes	Yes	Yes	Yes	Yes	Yes
Young 2018	Yes	Yes	Yes	Yes	Yes	Yes	Yes	*Can't tell	*Can't tell	*No	*No	*No	No	No	Can't tell	Can't tell	No*

Additional file V: Data extracted from the studies included in the systematic review

Data	Response options (if applicable)
Publication details: author, title, year	
Country; state/territory (if applicable)	
Aim of study	
Study design	• *Randomised controlled trial*<br>• *Non-randomised experimental study*<br>• *Cohort study*<br>• *Cross sectional study*<br>• *Case control study*<br>• *Qualitative research*<br>• *Multiple case study*<br>• *Case study*<br>• *Economic evaluation*<br>• *Other:________________*
**PARTICIPANTS**	
Participants, individuals	• *Store/canteen managers*<br>• *Store/canteen staff*<br>• *Health promotion practitioners*<br>• *Dietitians*<br>• *Policymakers*<br>• *Other:___________________*
Number of individual participants	
Participating food outlets/setting type(s)	• *School canteens*<br>• *Workplace canteens/restaurants*<br>• *Restaurants*<br>• *Grocery stores/supermarkets*<br>• *Food outlets in healthcare facilities*<br>• *Food outlets in sports and recreational centres*<br>• *Other:___________________*
Number of participating food outlets	

INTERVENTION DETAILS	
Intervention length (months)	
Healthy food retail intervention strategies used	• *Availability of healthy foods/drinks*<br>• *Availability of less healthy foods/drinks*<br>• *Placement/prominence*<br>• *Pricing strategies*<br>• *Promotional material or activity*<br>• *Consumer education*<br>• *Other:___________________*
Products targeted	• *Whole product offering*<br>• *Food only*<br>• *Drinks only*<br>• *Fruit and vegetables only*<br>• *Sugar-sweetened drinks only*<br>• *Other:___________________*
**IMPLEMENTATION**	
Implementation date (month / year)	
Implementation framework used (if applicable)	
**IMPLEMENTATION/SUSTAINMENT SUPPORT OFFERED**	
Support offered - either implementation or sustainment<br>*(e.g., training, monitoring)*	
Which of the following ERIC[1] categories is/are relevant to the support offered (if applicable)	• *Use evaluative and iterative strategies (e.g., audit and feedback, monitoring, needs assessments)*<br>• *Provide interactive assistance (e.g., supervision and technical assistance, tailored strategies and adaptations)*<br>• *Develop stakeholder interrelationships (e.g., involve governing structures/organisations in*

[1] Nathan N, Powell BJ, Shelton RC *et al.* (2022) Do the Expert Recommendations for Implementing Change (ERIC) strategies adequately address sustainment? *Frontiers in Health Services* 2.

	the implementation and sustainment, use working/advisory groups, identify program champions)<br>• *Train and educate stakeholders (e.g., educational materials, ongoing training/consultation, 'train the trainer')*<br>• *Support retail managers and staff (e.g., resource sharing)*<br>• *Engage consumers (e.g., increase demand for healthier foods, customer feedback and suggestions)*<br>• *Utilise financial strategies (e.g., external funding, incentivise the implementation and sustainment, disincentives for failure to sustain the intervention)*<br>• *Change infrastructure (e.g., physical structure and equipment, outlet location, mandate change)*<br>• *None of the above*<br>• *N/A*
**SUSTAINMENT**	
Data on sustainment factors collected (month / year)<br>E.g., date of interviews/surveys with retailers	
Evaluation framework used (if applicable)	
Data source(s)	• *Interview(s)*<br>• *Focus group(s)*<br>• *Survey(s)*<br>• *Observation(s)*<br>• *Other:___________________*
**Determinants of sustained healthy food retail changes – using the Integrated Sustainability Framework[2] adapted to food retail[3]**	

[2] Shelton RC, Cooper BR Stirman SW (2018) The Sustainability of Evidence-Based Interventions and Practices in Public Health and Health Care. *Annual Review of Public Health* 39, 55-76.
[3] Boelsen-Robinson T, Blake MR, Brown AD *et al.* (2021) Mapping factors associated with a successful shift towards healthier food retail in community-based organisations: A systems approach. *Food Policy* 101, 102032.

Outer contextual factors -quote(s)<br>*Please copy sections from the article that demonstrate findings related to this domain*	*[add quotes here]*
	Outer contextual factors in above quotes are related to...<br>• *Policy and legislation / sociopolitical context*<br>• *External recognition for healthy retail practice (e.g., media publications)*<br>• *Funding environment*<br>• *National/governmental leadership*<br>• *National/governmental values, priorities and needs*<br>• *Community ownership (broader population)*<br>• *Broader food environment (e.g., food industry and marketing)*<br>• *None*<br>• *Other:____________________*
Interorganisational factors -quote(s)<br>*Please copy sections from the article that demonstrate findings related to this domain*	*[add quotes here]*
Interorganisational factors in above quotes are related to...	• *Food suppliers/distributors*<br>• *Competition*<br>• *External partnerships, support and resources*<br>• *External funding*<br>• *Characteristics of the external governing organisation (if applicable)*<br>• *Other:____________________*
Inner contextual factors -quote(s)<br>*Please copy sections from the article that demonstrate findings related to this domain*	*[add quotes here]*

Inner contextual factors in above quotes are related to...	• *Funding / financial resources*<br>• *Material resources and equipment*<br>• *Organisational climate/culture*<br>• *Organisational stability/staffing turnover*<br>• *Characteristics of the food outlet/organisation*<br>• *Capacity (human resources and time)*<br>• *Champion (e.g. champion retailer or staff member)*<br>• *Mission and internal policies (alignment with healthy practices)*<br>• *Other:*_____________________
Processes -quote(s)<br>*Please copy sections from the article that demonstrate findings related to this domain*	*[add quotes here]*
Processes in above quotes are related to...	• *Partnerships and engagement*<br>• *Training, support and supervision*<br>• *Evaluation: fidelity, monitoring and reinforcement*<br>• *Evaluation and evidence: effectiveness (e.g., influence on population health)*<br>• *Adaptation*<br>• *Planning*<br>• *Team/board functioning*<br>• *Communication*<br>• *Technical assistance*<br>• *Capacity building*<br>• *Increasing or creating demand for healthier options*<br>• *Other:*_____________________
Characteristics of the retailer -quote(s)<br>*Please copy sections from the article that demonstrate findings related to this domain*	*[add quotes here]*

Characteristics of the retailer in above quotes are related to...	• *Retailer or store manager characteristics, motivation and willingness*<br>• *Retailer or store manager skills and expertise*<br>• *Food outlet staff characteristics, motivation and willingness*<br>• *Food outlet staff skills and expertise*<br>• *Other:_____________________*
Characteristics of the customers - quote(s)<br>*Please copy sections from the article that demonstrate findings related to this domain*	*[add quotes here]*
Characteristics of the customers in above quotes are related to...	• *Customer characteristics (e.g., demographics)*<br>• *Customer needs (perceived or actual)*<br>• *Customer motivation and purchasing behaviour*<br>• *Other:_____________________*
Characteristics of the intervention - quote(s)<br>*Please copy sections from the article that demonstrate findings related to this domain*	*[add quotes here]*
Characteristics of the intervention in above quotes are related to...	• *Adaptability*<br>• *Fit with population and context*<br>• *Influence on population health*<br>• *Profitability and/or cost*<br>• *Burden and complexity*<br>• *Trialability*<br>• *Other:_____________________*

Additional file VI: Factors identified to influence the sustainment of healthy food retail (HFR) environment changes, organised by Integrated Sustainability Framework[1] domains and factors

**Integrated Sustainability Framework (adapted to food retail)**			**Number of studies reporting each factor**	**Detailed findings for most frequently reported factors**
**Domain**	**Factor**	**Definition**		
**Outer contextual factors**			**11** [2-11]	
	**Sociopolitical environment (including policies and legislation)**	The broader sociopolitical context, including existing policies, regulations, guidelines, and mandates that may impact the sustainment of HFR practices.	6 [2-4,6,9,11]	State/nation-wide policies are good enforcement mechanisms but insufficient alone[3,9]<br>State/nation-wide policies can be effective in promoting long-term HFR changes[4,11]<br>State/nation-wide policies are too restrictive for some food outlets [9]<br>Other political regulations limit the ability to prioritise HFR practices[2]
	**Broader food environment (e.g., food market, food marketing, weather) ***	The general availability of healthy foods, influenced by factors such as natural resources, weather conditions, and agricultural subsidies, which impact the market supply. Additionally, the appeal of healthy options outside of food retail outlets, shaped by promotional methods used by stakeholders, including marketing strategies by food manufacturers.	5 [5,8-11]	Volatile food markets and fluctuating prices[8]<br>A significant change in the broader food environment and supply is required[11]<br>Limited availability of healthy products from food manufacturers[9,11]
	External recognition for healthy food retail practice *	External recognition for HFR changes made (e.g., accreditation/certification programs, media publications, etc.)	4 [5-7,10]	
	Community ownership	Members of the community leading efforts to promote HFR practices.	3 [2,4,7]	
	National / governmental leadership	Government leading HFR initiatives (e.g., government-led policy actions that target food retail practice).	3 [6,9,11]	

Integrated Sustainability Framework (adapted to food retail)			Number of studies reporting each factor	Detailed findings for most frequently reported factors
Domain	Factor	Definition		
	National values, priorities and broader population needs	The extent to which HFR practice is regarded as a national priority or fits with national, state, or local government priorities, needs, and values. For example, governmental prioritisation of HFR transformation to prevent diet-related diseases.	1 [6]	
	Government funding environment	The allocation of government budgets and the overall funding environment either support or hinder HFR practices.	0	
**Interorganisational factors ***			**15 [2-16]**	
	**External partners, support and resources***	Support and resources provided by external partners, such as local governments, health departments, health promotion agencies, universities and other supportive organisations. This includes non-financial resources provided by these stakeholders.	**12 [2-10,12-14]**	HFR practices are often not prioritised by food retailers, requiring sustainment to be driven by external partners focused on population health[3,6,7,10,12]<br><br>Partnerships facilitate ongoing support and resource- and knowledge-sharing [2,4-8,10,12-14]
	**Food supply and supplier related factors ***	Characteristics of food distributors/suppliers and availability, accessibility and affordability of healthy food options provided by distributors/suppliers to retailers.	**9 [5-11,13,14]**	Limited availability of healthy options from suppliers[5,8,9,11,13,14]<br><br>Suppliers offering healthy options have higher prices[5,8,13]
	**Competition***	Availability of other food outlets, their product offerings, and their locations, which may create competition among food outlets.	**5 [3,8,9,11,16]**	Smaller food outlets are at a disadvantage compared to larger grocery stores and supermarkets due to their size, pricing challenges, and limited purchasing power[8,9,16]<br><br>HFR policies and practices are less effective and unfair when customers could easily access cheaper, less healthy alternatives at nearby food outlets[3,8,9,11,16]
	External funding *	Funding and financial support provided by external stakeholders or governing organisations (e.g., health departments, health promotion agencies, local governments/councils, other governing organisations) to support implementation and/or sustainment of HFR changes.	4 [2,6,10,16]	

Integrated Sustainability Framework (adapted to food retail)			Number of studies reporting each factor	Detailed findings for most frequently reported factors
**Domain**	**Factor**	**Definition**		
	Governing organisations *(if applicable)* *	The motivation, willingness, and operational capacity of governing bodies or external organisations (e.g., local governments that own or oversee food outlets in public settings) to drive and sustain HFR changes. *Mainly applicable to food outlets in public settings.*	4[2,3,11,15]	
	Contractual requirements *	Healthy food retail practices are required as part of long-term rental contracts or other agreements with retailers in public settings.	2 [3,15]	
**Characteristics of customers**			**10 [2,5-8,10-13,16]**	
	**Customer motivation and purchasing behaviour**	General healthiness of customer purchases and customers' response to healthy changes in the food retail outlet.	**10 [2,5-8,10-13,16]**	If customers are not interested in the healthy food products, food retailers are unlikely to maintain these items in their product offerings[2,5-8,10-13,16]
	**Customer characteristics**	Customer demographics, socioeconomic status etc.	**5 [8,10-12,16]**	Low-income customers face financial constraints, prioritising value for money (e.g., low-cost energy-dense options, large portions) while struggling to afford healthier foods [8,12,16]<br>Comfort food can be a coping mechanism in high-stress environments[11]
	Customer needs	Perceived or actual need for healthier food options within a community.	3 [8,11,12]	
**Inner contextual factors**			**12 [2,3,5-12,15,16]**	
	**Characteristics of the food outlet (business)***	This includes factors such as the type of food outlet (e.g., supermarket, small store, cafe), its location, branding or organisational identity (e.g., whether it identifies as a "healthy food outlet"), and its organisational structure (e.g., independently owned vs. part of a larger chain, number of employees, etc.).	**7 [5,8-12,16]**	Food outlet type and characteristics influence customer expectations about available products, influencing food retailers' willingness to stock certain items.[8,10,12,16]

Integrated Sustainability Framework (adapted to food retail)			Number of studies reporting each factor	Detailed findings for most frequently reported factors
Domain	Factor	Definition		
	**Material resources**	Availability and condition of material resources required to sustain the HFR changes. This includes appropriate equipment such as operational refrigerators for storing fresh, healthy foods.	**6** [5-9,11]	Equipment damage, maintenance costs, and misuse for less healthy products hinder long-term HFR changes[5,6]<br><br>Regular updates on supporting resources and promotional materials are needed to maintain their relevance over time[7,11,16]
	Internal funding and financial resources	Internal funding available and/or allocated for implementing and sustaining healthy changes in the product offering.	4 [2,5,7,10]	
	Organisational culture and climate	General organisational climate/culture is supportive of HFR practice.	4 [2,3,7,15]	
	Capacity (human resources and time)	Availability of human resources and time required to sustain healthy changes in food outlets. For example, availability of staff with the relevant nutritional knowledge/experience needed to identify healthy and less healthy foods.	3 [2,6,7]	
	Mission and internal policies (alignment with healthy practices)	Healthy food retail practices are aligned with the organisation's mission, strategies, plans, and policies. For example, promoting HFR practices is a priority within the organisation's overarching goals and objectives.	3 [3,11,15]	
	Organisational stability / staffing turnover	Stability of the organisation, including staff attrition and turnover rates, changes in leadership, and organisational restructuring, which can all influence the sustainment of HFR practices.	1[7]	
	Champion	Food retail organisations or individuals that have strong influences on other retailers or food outlet staff and promote the ongoing delivery of HFR practices.	0	
**Characteristics of the retailer (interventionist)**			**9** [3,5-8,10,11,13,16]	
	**Retailer and/or store manager characteristics,**	Retailer and/or store manager's motivation and willingness to sustain healthy changes. For example, store manager's support and perceived risks and benefits of the HFR intervention.	**9** [3,5-8,10,11,13,16]	Lack of motivation or incentives to maintain healthy changes hinders sustainment efforts[3,11,16], while motivation can support these[5,8,10,16]

Integrated Sustainability Framework (adapted to food retail)			Number of studies reporting each factor	Detailed findings for most frequently reported factors
Domain	Factor	Definition		
	**motivation and attitudes**	This can be influenced by retailer's/ manager's previous (successful or unsuccessful) experience with healthy food initiatives.		Retailer and/or store manager motivation alone is not sufficient, as other factors (e.g., competing priorities, competition between food outlets, financial trade-offs) may hinder sustainment efforts.[3,5,7,8,10,11,16]
	Food outlet staff characteristics, motivation and attitudes	Food outlet staff's motivation and willingness to sustain healthy changes.	2 [6,8]	
	Retailer and/or store manager knowledge, skills and expertise	Retailer/store manager's skills and knowledge required to sustain HFR practice. For example, if a retailer has significant experience in successfully implementing and sustaining HFR initiatives, it suggests that they have likely developed expertise in this area.	2 [5,10]	
	Food outlet staff's knowledge, skills and expertise	Food outlet staff's skills and knowledge required to implement and sustain HFR practice, including previous experience with HFR interventions.	0	
**Characteristics of the intervention**			**12** [2,4-9,11,13-16]	
	**Profitability and cost**	Perceived and actual financial benefits and costs associated with maintaining healthy changes in food retail outlet (e.g., new revenue stream, food waste from fresh food).	**11** [2,5-13,16]	Maintenance of HFR changes causes additional costs (e.g., food waste, higher expenses related to obtaining and stocking healthy foods)[5-11,13,16]<br><br>Healthy options are less profitable compared to less healthy options[5,6,8-13]<br><br>Healthy options can be a new revenue stream[5]
	**Burden and complexity**	Retail outlet staff, manager, and/or owner time and resources required to implement and sustain healthy changes in the food outlet. This includes considering the complexity of the	**10** [5-11,13,14,16]	Maintenance of HFR changes requires additional time and resources[5,7-9,13,14,16]

Integrated Sustainability Framework (adapted to food retail)			Number of studies reporting each factor	Detailed findings for most frequently reported factors
Domain	Factor	Definition		
		intervention components, workload and financial investments required from staff, managers, or owners to support and sustain these changes over time.		
	**Fit with population and context**	Fit of a healthy food intervention within a context, populations (customers), and retail organisations. This refers to how well the intervention aligns with the specific characteristics and needs of the community, the target customer base, and the operational structure of the retail organisations.	**9** [2,6,8-12,15,16]	Intervention components and support should be tailored to the specific context of the food outlet [2,6,8-12,15,16]
	Intervention components *	The individual elements or strategies within an intervention that may vary in their ease of sustainment. Some components might be simpler to maintain over time due to factors like lower resource demands, higher perceived value, or ease of integration into existing systems, while others might require more effort, support, or resources to sustain effectively.	4 [5-7,16]	
	Adaptability	Degree to which a HFR intervention can be tailored or refined to fit different settings or population needs.	4 [6-8,16]	
	Influence on population health	Perceived and actual influence/benefits of the healthy changes in food retail outlet on population health outcomes.	1[8]	
	Trialability	Ease with which a HFR intervention can be tested on a smaller scale before full implementation.	0	
**Processes**			**15** [2-16]	
	**Fidelity, monitoring and enforcement**	Processes aimed at maintaining fidelity to intervention components (e.g., through monitoring and auditing) and ensuring accountability of food outlets.	**9** [2,3,6,9-12,15,16]	Ongoing monitoring and reinforcement activities are essential for sustained changes[2,3,6,9-12,15,16]
	**Partnership and engagement**	Establishing and maintaining partnerships with key stakeholders, such as local governments, health departments, health promotion agencies, universities and other supportive organisations, to support and sustain healthy changes. This includes ongoing engagement with key stakeholders.	**8** [2,4,6-9,11,16]	Establishing and maintaining connections with key external partners (e.g., healthy food suppliers, health promotion agencies, public health professionals, community groups) helps maintain momentum and address other determinants of sustained changes (e.g., resources, profitability)[2,4,6-9,11,16]

Integrated Sustainability Framework (adapted to food retail)			Number of studies reporting each factor	Detailed findings for most frequently reported factors
Domain	Factor	Definition		
	**Technical assistance, support and supervision**	Processes employed by external stakeholders to offer technical support and/or supervision for sustaining HFR practices in food outlets. This may involve providing practical support (e.g. updating lists of available healthy product options) as well as regular contact and site visits.	**8** [6,7,10-15]	Retailers often view supervision from external partners as essential for sustaining HFR practices[6,7,10,11,13], whereas public health professionals sometimes believe retailers should learn to sustain these practices independently[10]
	**Increasing or creating consumer demand for healthy options** *	Processes employed by the retailer or external stakeholders to increase or create demand for healthy foods and drinks. For example, improving the appeal of the store and promotion of healthy food and drinks.	**7** [4-8,10,14]	Promoting healthy options within (e.g., promotional materials and pricing strategies) and outside food outlets (e.g., through media) is key to generating customer demand and encouraging food retailers to maintain healthy products in their offerings[5-8,10,14]
	**Communication**	Internal communication: processes to ensure that retail outlet staff are informed about the initiative and new practices.<br>External communication: communication between retailers and stakeholders supporting the initiative.	**6** [2,4-6,10,16]	Ongoing communication between retailers and external partners (e.g., public health professionals, health promotion agencies) helps retailers address challenges and clarify expectations[2,6,10,16], while a lack of communication can hinder sustained changes[10,16]
	Training	Processes that can influence retail manager and staff knowledge, skills, and motivation to implement and sustain healthy changes in the retail outlet.	3 [6,7,11]	
	Capacity building	Activities/processes that are aimed at empowering food retailers to sustain HFR practices over time. These activities aim to provide retailers with the necessary skills, knowledge, and resources to continue offering healthy food options effectively.	2 [6,7]	
	Planning	Ongoing sustainment of the HFR changes is planned.	0	
	Adaptation	Processes to actively and systematically guide adaptation of a HFR initiative to better fit the target food outlets.	0	
	Evaluation and evidence: effectiveness	Collecting and presenting evidence to guide the planning and decision-making processes for HFR interventions. For example, using available evidence to develop intervention strategies that are grounded in research and likely to result in sustained changes. Additionally, this includes offering evidence-based feedback to food outlets to illustrate the effects of the healthy changes implemented in their product offerings.	0	

Integrated Sustainability Framework (adapted to food retail)			Number of studies reporting each factor	Detailed findings for most frequently reported factors
Domain	Factor	Definition		
	Team/board functioning	Sustainment processes are integrated as part of existing food outlet practices.	0	

HFR = Healthy food retail

*New domain or factor identified through the data synthesis process

Factors cited by ≥5 sources are highlighted in **bold.**

References

1. Shelton RC, Cooper BR, Stirman SW. The sustainability of evidence-based interventions and practices in public health and health care. *Annual Review of Public Health*. 2018;39(1):55-76. doi:10.1146/annurev-publhealth-040617-014731

2. Greaney ML, Hardwick CK, Spadano-Gasbarro JL, et al. Implementing a multicomponent school-based obesity prevention intervention: a qualitative study. *Journal of nutrition education and behavior*. 2014;46(6):576-582. doi:10.1016/j.jneb.2014.04.293

3. Levay AV, Chapman GE, Seed B, Wittman H. District-level implementation of British Columbia's school food and beverage sales policy: a realist evaluation exploring intervention mechanisms in urban and rural contexts. *Canadian Journal of Public Health*. 2019;110(1):21-30. doi:10.17269/s41997-018-0159-x

4. Pettigrew S, Talati Z, Sauzier M, Ferguson A. Stakeholder perceptions of a school food policy ten years on. *Public Health Nutrition*. 2018;21(7):1370-1374. doi:10.1017/S1368980017003962

5. Boys KA, Haynes-Maslow L, McGuirt JT, et al. Perceived barriers and facilitators to participating in the North Carolina Healthy Food Small Retailer Program: a mixed-methods examination considering investment effectiveness. *Public health nutrition*. 2021;24(18):6555-6565. doi:10.1017/S1368980021003955

6. Gittelsohn J, Laska MN, Karpyn A, Klingler K, Ayala GX. Lessons learned from small store programs to increase healthy food access. *American Journal of Health Behavior*. 2014;38(2):307-315. doi:10.5993/AJHB.38.2.16

7. Vargas C, Whelan J, Feery L, et al. Developing co-creation research in food retail environments: a descriptive case study of a healthy supermarket initiative in regional Victoria, Australia. Article. *International Journal of Environmental Research and Public Health*. 2023;20(12)doi:10.3390/ijerph20126077

8. Lynch M, Graham M, Taylor K, Mah CL. Corner store retailers' perspectives on a discontinued healthy corner store initiative. *Community Health Equity Research & Policy*. 2023;43(4):421-429. doi:10.1177/0272684X211004930

9. McGuire CM, Winkler MR, Lenk KM, Harnack L, Erickson DJ, Laska MN. Small food store retailers' views on healthy food retail policy in response to a local staple foods ordinance. *Public Health Nutrition*. 2021;24(7):1934-1940. doi:10.1017/S1368980020000580

10. Lynch M, Giles A. Using diffusion of innovations theory to examine a discontinued heathy corner store pilot program. *Journal of Hunger & Environmental Nutrition*. 2024/05/03 2024;19(3):313-328. doi:10.1080/19320248.2022.2105186

11. Rosin M, Ni Mhurchu C, Mackay S. Implementing healthy food policies in health sector settings: New Zealand stakeholder perspectives. *BMC Nutrition*. 2024/09/07 2024;10(1):119. doi:10.1186/s40795-024-00924-z

12. Boelsen-Robinson T, Peeters A, Thow A-M, Hawkes C. Barriers and facilitators to implementing a healthier food outlet initiative: perspectives from local governments. *Public Health Nutrition*. 2021;24(9):2758-2770. doi:10.1017/S1368980020002323

13. Law KK, Pulker CE, Healy JD, Pollard CM. "Just so you know, it has been hard": food retailers' perspectives of implementing a food and nutrition policy in public healthcare settings. *Nutrients*. 2021;13(6):2053-2053. doi:10.3390/nu13062053

14. Young S, DeNomie M, Sabir J, Gass E, Tobin J. Around the Corner to Better Health: A Milwaukee corner store initiative. *American Journal of Health Promotion*. 2018;32(6):1353-1356. doi:10.1177/0890117117736970

15. Wood M, Robles B, Beltran J, Kuo T. Integrating healthy nutrition standards and practices Into food service contracting in a large US county government. *Prev Chronic Dis*. Mar 7 2024;21:E15. doi:10.5888/pcd21.230220

16. Adams J, Halligan J, Watson DB, et al. The change4life convenience store programme to increase retail access to fresh fruit and vegetables: A mixed methods process evaluation. Article. *PLoS ONE*. 2012;7(6)doi:10.1371/journal.pone.0039431
